# Highly Mutagenic Exocyclic DNA Adducts Are Substrates for the Human Nucleotide Incision Repair Pathway

**DOI:** 10.1371/journal.pone.0051776

**Published:** 2012-12-14

**Authors:** Paulina Prorok, Christine Saint-Pierre, Didier Gasparutto, Olga S. Fedorova, Alexander A. Ishchenko, Hervé Leh, Malcolm Buckle, Barbara Tudek, Murat Saparbaev

**Affiliations:** 1 Institute of Biochemistry and Biophysics, Polish Academy of Sciences, Warsaw, Poland; 2 Groupe «Réparation de l’ADN», CNRS UMR8200, Université Paris-Sud, Institut de Cancérologie Gustave Roussy, Villejuif, France; 3 Laboratoire Lésions des Acides Nucléiques, SCIB/UMR E3 CEA-UJF, INAC, CEA, Grenoble, France; 4 Institute of Chemical Biology and Fundamental Medicine, Siberian Branch of the Russian Academy of Sciences, Novosibirsk, Russia; 5 LBPA, ENS de Cachan, CNRS, Cachan, France; 6 Institute of Genetics and Biotechnolgy, University of Warsaw, Warsaw, Poland; University of Massachusetts Medical School, United States of America

## Abstract

**Background:**

Oxygen free radicals induce lipid peroxidation (LPO) that damages and breaks polyunsaturated fatty acids in cell membranes. LPO-derived aldehydes and hydroxyalkenals react with DNA leading to the formation of etheno(ε)-bases including 1,*N* ^6^-ethenoadenine (εA) and 3,*N* ^4^-ethenocytosine (εC). The εA and εC residues are highly mutagenic in mammalian cells and eliminated in the base excision repair (BER) pathway and/or by AlkB family proteins in the direct damage reversal process. BER initiated by DNA glycosylases is thought to be the major pathway for the removal of non-bulky endogenous base damage. Alternatively, in the nucleotide incision repair (NIR) pathway, the apurinic/apyrimidinic (AP) endonucleases can directly incise DNA duplex 5′ to a damaged base in a DNA glycosylase-independent manner.

**Methodology/Principal Findings:**

Here we have characterized the substrate specificity of human major AP endonuclease 1, APE1, towards εA, εC, thymine glycol (Tg) and 7,8-dihydro-8-oxoguanine (8oxoG) residues when present in duplex DNA. APE1 cleaves oligonucleotide duplexes containing εA, εC and Tg, but not those containing 8oxoG. Activity depends strongly on sequence context. The apparent kinetic parameters of the reactions suggest that APE1 has a high affinity for DNA containing ε-bases but cleaves DNA duplexes at an extremely slow rate. Consistent with this observation, oligonucleotide duplexes containing an ε-base strongly inhibit AP site nicking activity of APE1 with IC_50_ values in the range of 5–10 nM. MALDI-TOF MS analysis of the reaction products demonstrated that APE1-catalyzed cleavage of εA•T and εC•G duplexes generates, as expected, DNA fragments containing 5′-terminal ε-base residue.

**Conclusions/Significance:**

The fact that ε-bases and Tg in duplex DNA are recognized and cleaved by APE1 *in vitro*, suggests that NIR may act as a backup pathway to BER to remove a large variety of genotoxic base lesions in human cells.

## Introduction

**Figure 1:**
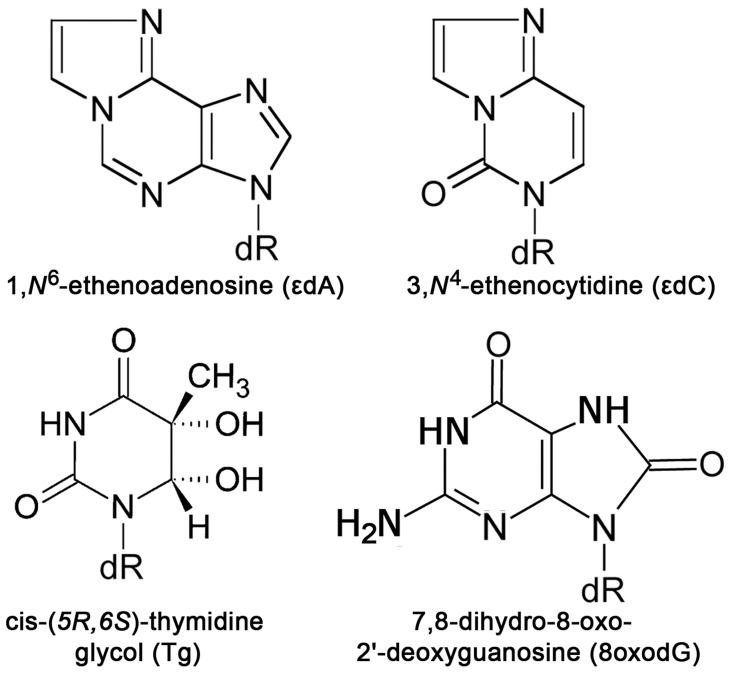
The chemical structures of ε-adducts and oxidized bases.

The etheno (ε) ring system is formed by the attack of reactive bi-functional epoxides or aldehydes at the exocyclic nitrogen atoms of DNA bases, followed by dehydration and ring closure [1], [2]. The ε-derivatives of DNA bases such as 1,*N* ^6^-ethenoadenine (εA) and 3,*N* ^4^-ethenocytosine (εC) (Figure 1) are generated in cellular DNA either by reaction with epoxides that result from the metabolism of various industrial pollutants such as vinyl chloride (VC) or via endogenous processes through the interaction of lipid peroxidation (LPO)-derived aldehydes and hydroxyalkenals [3]. The ε-adducts are ubiquitous and have been found in DNA isolated from tissues of untreated rodents and healthy human subjects [4]. However, εA and εC levels were significantly increased by cancer risk factors contributing to oxidative stress/LPO, such as chronic infections and inflammatory conditions [5]. The deleterious effects of ε-bases are due to the mutagenic potential of these exocyclic DNA lesions. During DNA replication in *E. coli* and simian kidney cells, εC mostly produces εC•G to A•T transversions and εC•G to T•A transitions [6], [7]. In a single-stranded shuttle vector containing a single εC residue, the targeted mutation frequency yield was 2% in *E. coli*, 32% in SOS-induced *E. coli* cells, and 81% in simian kidney cells [7]. The εA residues are highly mutagenic in mammalian cells, where they mainly lead to εA•T to T•A transitions, but are weakly mutagenic in *E. coli* [8], [9].

Most endogenously generated DNA base lesions are removed by DNA glycosylases in the base excision repair pathway (BER). A DNA glycosylase excises the modified base, leaving, as an end product, either an apurinic/apyrimidinic (AP) site or a single-stranded DNA break with 3′-sugar phosphate groups that must be removed prior to the gap-filling synthesis step [10], [11]. It has been established that εA and εC are recognized and excised by human mono-functional DNA glycosylases: alkyl-*N*-purine-DNA glycosylase (ANPG/Aag/MPG) and mismatch-specific thymine-DNA glycosylase (TDG), respectively [12], [13], [14]. Surprisingly, APNG/Aag null mice do not exhibit any particular sensitivity to vinyl carbamate, an environmental carcinogen, although higher levels and increased persistence of εA residues were observed in hepatic DNA from *APNG* ^−/−^ mice after exposure to this agent [15]. These observations suggest the existence of alternative repair pathways for ε-bases in mammalian cells. Indeed, it has been demonstrated that εA and εC are eliminated from DNA in the direct damage reversal repair pathway *via* oxidative dealkylation catalyzed by AlkB-family proteins. These enzymes remove alkyl groups by direct reversal of alkylated bases to normal ones without DNA cleavage and *de novo* DNA synthesis [16], [17]. Recent studies demonstrated that the human AlkB homologue ABH2, removes εA and εC residues from DNA in mammalian cells [18], [19]. Nevertheless, repair efficiency of ABH2 towards εA was not sufficient to compensate the lack of BER leading to accumulation of εA residues in the genome of 24 months old *APNG^−/−^* mice [18].

Intriguingly, genetic data demonstrate that mice mutants lacking DNA glycosylases are not sensitive to DNA damaging agents, pointing to redundancy in the repair pathways [20]. In the alternative nucleotide incision repair (NIR) pathway, an AP endonuclease makes an incision 5′ next to a damaged base in a DNA glycosylase-independent manner, resulting in a genuine 3′-OH group for DNA polymerization and a 5′-dangling damaged nucleotide [21]. While the general assumption that BER, initiated by multiple DNA glycosylases, is the main pathway for removal of the majority of oxidized bases [22], [23], certain types of lesions such as the alpha-anomers of 2′-deoxynucleosides (αdN) are not repaired by DNA glycosylases but rather by AP endonucleases in the NIR pathway [24], [25], [26]. Furthermore, oxidatively damaged pyrimidines including 5,6-dihydrothymine (DHT), 5,6-dihydrouracil (DHU), 5-hydroxyuracil (5OHU), 5-hydroxycytosine (5OHC), 5-hydroxyhydantoin (5OH-Hyd) and 5-hydroxy-5-methylhydantoin (5OH-5Me-Hyd) are substrates for both the BER and NIR pathways suggesting redundancy in the repair of oxidative base lesions [21], [26], [27], [28].

In human cells, the major AP endonuclease 1 (APE1/Ref-1/HAP-1) initiates NIR pathway by cleaving duplex DNA 5′ next to oxidatively damaged bases [26]. APE1 was independently discovered as an abasic site-specific endonuclease homologous to the *E. coli* Xth protein [29] and as a redox-regulator of the DNA binding domain of Fos-Jun, Jun-Jun, AP-1 proteins and several other transcription factors [30]. In addition to AP endonuclease and NIR activities, APE1 exhibits other critical DNA repair activities: 3′→5′ exonuclease, 3′-phosphodiesterase, 3′-phosphatase and RNase H [29]. Divalent cation, pH and ionic strength requirements of the APE1-catalyzed *versus* AP endonuclease catalyzed NIR activity are dramatically different. At low concentrations of Mg^2+^ (≤1 mM) APE1 exhibits dramatically increased 3′→5′ exonuclease [31] and NIR-endonuclease [26] activities. Interestingly, independent measurements of the concentration of intracellular free Mg^2+^ in human brain, platelets, lymphocytes and skeletal muscle using different approaches gave values of less than 1 mM [32] supporting our previous observation that an intracellular environment can maintain APE1-catalyzed NIR function [33]. Furthermore, various DNA repair functions of APE1 can be separated by constructing mutants deficient in the NIR activity but still capable of performing the BER functions [34].

Despite the observed accumulation of endogenous DNA damage in single *ANPG^−/−^*, *mNth1^−/−^*, *mOGG1^−/−^* and *Neil1^−/−^* DNA glycosylase deficient mice, they do not exhibit cancer-prone phenotypes suggesting the existence of back-up repair pathways [20]. Previously, we have demonstrated that the AP endonucleases of *E. coli* Nfo, yeast Apn1 and human APE1 initiate the NIR pathway by incising the duplex DNA containing various oxidatively damaged bases implying a potential role of this mechanism as a back-up system to classic BER. Here, we demonstrate that human APE1 cleaves 5′ next to ε-bases and thymine glycol residues when present in the duplex DNA substrate. Matrix Assisted Laser Desorption Ionisation Time-Of-Flight (MALDI-TOF) Mass Spectrometry (MS) analysis of the APE1 cleavage products, unambiguously confirmed the formation of a DNA fragment containing the respective 5′-dangling damaged base. The potential biological importance of the reported new substrate specificity of APE1 in cleansing genomic DNA of potentially mutagenic and cytotoxic lesions is discussed.

## Materials and Methods

### Oligonucleotides and Proteins

Sequences of the specifically modified deoxyribo-oligonucleotides used in the present work are shown in Table 1. The 19 mer oligonucleotide containing 5,6-dihydroxy-5,6-dihydrothymidine (or thymine glycol) (Tg) was kindly provided by Hiroshi Ide (Hiroshima University, Japan) [35]. All other oligonucleotides were purchased from Eurogentec (Seraing, Belgium) including those containing εA, εC, tetrahydrofuran (THF, synthetic AP site), αdA, Tg and 7,8-dihydro-8-oxoguanine (8oxoG) residues and complementary oligonucleotides, containing either dA, dG, dC or T opposite to the adduct (see Table 1). 22 mer oligodeoxyribonucleotides X22, d(CACTTCGGAXTGTGACTGATCC), where X is either εA, εC, THF, αdA or 8oxoG, and complementary N22, d(GGATCAGTCACANTCCGAAGTG), where N is either dA, dG, dC or T were hybridized to obtain duplexes referred to as X22•N. This sequence context was previously used to study the repair of pyrimidine-derived hydantoines [28]. Prior to enzymatic assays, oligonucleotides were either 3′-end labelled by terminal deoxynucleotidyl transferase (New England Biolabs France) in the presence of [α-^32^P]-3′-dATP (Cordycepin 5′-triphosphate, 5,000 Ci/mmol^-1^) or 5′-end labelled by T4 polynucleotide kinase (New England Biolabs) in the presence of [γ-^32^P]-ATP (3,000 Ci/mmol-1) (PerkinElmer, Life Science Research, Courtaboeuf, France), as recommended by the manufacturers. Radioactively labelled oligonucleotides were desalted with a Sephadex G-25 column equilibrated in water and then annealed with corresponding complementary strands for 3 min at 65°C in a buffer containing 20 mM HEPES-KOH (pH 7.6), 50 mM KCl.

**Table 1:** Sequences of oligonucleotides bearing a single base lesion used to identify the NIR activity and relative efficiency of the APE1-catalyzed cleavage of duplex DNA substrates.

Name^a^	Sequence^b^	Lesion position	Sequence context	Cleavage efficiency^c^
1. εA-DL10	d(AATTGCTATXTAGCTCCGCACGCTGGTACCCATCTCATGA)	10	*TAT*X*TAG*	14.1%
2. εA-PP	d(CATCTCATGAAATTGCTATXTAGCTCCGCACGCTGGTACC)	20	*TAT*X*TAG*	9.7%
3. εN22	d(CACTTCGGAXTGTGACTGATCC)	10	*GGA*X*TGT*	7%
4. εN-C21	d(GCTCTCGTCTGXACACCGAAG)	12	*CTG*X*ACA*	4.2%
5. εN-RT	d(TGACTGCATAXGCATGTAGACGATGTGCAT)	11	*ATA*X*GCA*	11.7%
6. εN-DL	d(AATTGCTATCTAGCTCCGCXCGCTGGTACCCATCTCATGA)	20	*CGC*X*CGC*	None^d^
7. εA28	d(CAGCTCTGTACXTGAGCGGTGGTGACAC)	12	*TAC*X*TGA*	None
8. εN-MS	d(AAATACATCGTCACCTGGGXCATGTTGCAGATCC)	20	*GGG*X*CAT*	None
9. εN-PN	d(GGCTTCATCGTTATTXATGACCTGGTGGATACCG)	16	*ATT*X*ATG*	None
10. Tg-RT	d(TGACTGCATAYGCATGTAGACGATGTGCAT)	11	*ATA*X*GCA*	2.3%
11. Tg-IW	d(ACAGACGCCAYCAACCAGG)	11	*CCA*X*CAA*	3.9%
12. 8oxoG-RT	d(TGACTGCATAZGCATGTAGACGATGTGCAT)	11	*ATA*X*GCA*	None
13. 8oxoG22	d(CACTTCGGAZTGTGACTGATCC)	10	*GGA*X*TGT*	None
14. THF-22^e^	d(CACTTCGGAPTGTGACTGATCC)	10	*GGA*X*TGT*	>90%

a εN is either εA or εC.

b X is the position of ε-base, Y is the position of thymine glycol, Z is the position of 8-oxoguanine, P is the position of a synthetic AP site.

c Cleavage efficiency was expressed as the percentage of incision product produced after 2 h incubation at 37°C in the presence of 10 nM DNA substrate and 10 nM of APE1 under NIR conditions.

d Non-zero background levels of activity in non-treated oligonucleotides were subtracted when calculating APE1 activities. Background levels were varied from 0.3 to 2% and were due to non-specific spontaneous degradation and/or impurities of oligonucleotides.

e THF, 3-hydroxy-2-hydroxymethyltetrahydrofuran or tetrahydrofuran, is a synthetic analogue of an AP site. To measure cleavage efficiency a solution of 1 nM of 22 mer THF•T duplex oligonucleotide was incubated with 0.5 nM APE1 for 5 min at 37°C under NIR conditions.

The purified DNA glycosylases, AP endonucleases and human FEN1 were from the laboratory stock, prepared as described [34]. The purified human POLβ was purchased from Trevigen (Gaithersburg, USA).

### DNA Repair Assays

The standard reaction mixture (20 µL) contained 10 nM of [^32^P]-labelled εA•T and εC•G oligonucleotide duplexes and 5 nM of the purified APE1 protein and incubated for 2 h at 37°C, unless otherwise stated. The DNA repair activities of APE1 protein were tested either in the “NIR” buffer, which is optimal for the nucleotide incision activity and contained 50 mM KCl, 20 mM HEPES-KOH (pH 6.9), 0.1 mg•mL^−1^ BSA, 1 mM DTT and 0.1 mM MgCl_2_ or in the “BER+Mg^2+^” buffer, optimal for the AP endonuclease activity, containing 100 mM KCl, 20 mM HEPES-KOH (pH 7.6), 0.1 mg•mL^−1^ BSA, 1 mM DTT and 5 mM MgCl_2_. When measuring APE1-catalyzed NIR activity in human cell-free extracts MgCl_2_ was replaced with ZnCl_2_ [27]. The reaction mixture (20 µL) for the *E. coli* Nfo protein contained 50 mM KCl, 20 mM HEPES-KOH (pH 7.6), 0.1 mg•mL^−1^ BSA and 1 mM DTT and the same buffer but supplemented with 5 mM MgCl_2_ was used for the *S. cerevisiae* Apn1 protein.

The release of modified bases by the various DNA glycosylases was determined as a function of the amount of cleaved oligonucleotide containing a single base lesion at a defined position. The activities were measured in the “BER+EDTA” buffer containing 10 nM of duplex oligonucleotide substrate, 50 mM KCl, 20 mM HEPES-KOH (pH 7.6), 0.1 mg•mL^−1^ BSA, 1 mM DTT, 1 mM EDTA and 10 nM of a given purified protein. The reaction mixtures were incubated for 10 min at 37°C, unless otherwise stated. For the monofunctional DNA glycosylases, the abasic sites left after excision of the damaged bases were cleaved either by light piperidine treatment [10% (v/v) piperidine at 37°C for 30 min] or by APE1 in “BER+Mg^2+^” buffer. The reactions were stopped by adding 10 µL of stop solution containing 0.5% SDS and 20 mM EDTA, then desalted by hand-made spin-down columns filled with Sephadex G25 (Amersham Biosciences) equilibrated in 7.5 M urea. Purified reaction products were separated by electrophoresis in denaturing 20% (w/v) polyacrylamide gels (7 M urea, 0.5×TBE, 42°C). Gels were exposed to a Fuji FLA-3000 Phosphor Screen and analyzed using Image Gauge V3.12 software.

To measure kinetic parameters, the duplex oligonucleotide substrates (concentrations varied from 0.1 to 10 times the K_M_ generally from 0.5 nM to 50 nM) were incubated in the presence of limiting amount of enzyme (5 nM APE1) for 2 h at 37°C under NIR condition. For *K*_M_ and *k*_cat_ determination, the linear velocities were plotted against substrate concentration and the hyperbolic curve obtained fit to a rectangular hyperbola by least-squares non-linear regression method. Apparent values were obtained for the Michaelis constant, *K*_M_, and the *V*_max_ for cleavage; *k*_cat_ was calculated by dividing the *V*_max_ by the enzyme concentration. At least three independent experiments were performed for all kinetic measurements.

### *In vitro* Reconstitution of the NIR and BER Pathways

The *in vitro* reconstitution of the NIR pathway for εA and αdA residues was carried out in the presence of ATP that decreases the actual concentration of free Mg^2+^ in the reaction mix due to chelation of divalent cations. Briefly, 10 nM of non-labelled 40 mer εA-PP•T or 34 mer αdA•T oligonucleotide duplex was incubated either for 3 h or 1 h at 37°C, respectively, in the presence of 10 nM APE1, 2 nM FEN1, 0.02 U POLβ and 20 U T4 DNA ligase in the reaction buffer containing 50 mM HEPES-KOH (pH 7.2), 30 mM NaCl, 3 mM MgCl2, 2 mM ATP, 0.1 mg/ml BSA, 2 mM DTT, 5 mCi of [α-^32^P]dATP and 50 µM each of dGTP, dCTP and dTTP. Kinetics of the *in vitro* reconstitution of the NIR pathway for εA and αdA was performed in the same reaction buffer for varied time ranging from 0 to 120 min at 37°C in the presence of 10 nM APE1, 2 nM FEN1, 0.01 U POLβ and 4 U T4 DNA ligase. The *in vitro* reconstitution of the BER pathway for εA residues was carried out in the presence of the ANPG protein. Briefly, 10 nM of non-labelled 40 mer εA-PP•T oligonucleotide duplex was incubated for varied time ranging from 0.5 to 120 min at 37°C in the presence of 40 nM ANPG, 5 nM APE1, 2 nM FEN1, 0.01 U POLβ and 4 U T4 DNA ligase in the reaction buffer containing 20 mM HEPES-KOH (pH 7.6), 50 mM KCl, 5 mM MgCl_2_, 2 mM ATP, 0.1 mg/ml BSA, 1 mM DTT and 5 mCi of [α-^32^P]dATP. The reactions were stopped and products were analyzed as described above.

### Electrophoretic Mobility Shift Assay (EMSA)

The standard binding reaction mixture (20 µl) contained 20 mM Hepes-KOH, pH 7.6, 50 mM KCl, 10 µM or 100 µM MgCl_2_, 10 nM of 22 mer 3′-[^32^P]-labelled εA22•T, εC22•G, A22•T or C22•G and 250 nM or 500 nM APE1. The mixture was incubated for 10 min on ice, after which an aliquot, with addition of glycerol to final concentration 10%, was removed and analyzed by electrophoresis on a 8% non-denaturing polyacrylamide gel (80∶1 acrylamide/bisacrylamide) an electrophoresis buffer containing 6 mM Tris-HCl, pH 7.8, 5 mM sodium acetate and 0.1 mM EDTA at 160V for 14 h at +4°C. The gels were visualized as described above.

### Surface Plasmon Resonance Imagery (SPRi) Measurements of APE1 Interactions with DNA

Surface Plasmon Resonance imagery (SPRi) measurements were performed using SPRi-Plex™ apparatus (HORIBA Scientific) as previously described [36]. Thiolated single-stranded DNA oligonucleotides each containing a mercapto-hexane required for chemisorption on gold surfaces and five thymines - (T)_5_ as a spacer at the 5′ end were purchased from Eurogentec (Seraing, Belgium). The 22 mer thiolated single-stranded DNA oligonucleotide 5′-[Thiol-C6]-d(TTTTT-GGATCAGTCACATTCCGAAGTG) referred to as T22 was hybridized with complementary 22 mer εA22, A22 and THF-22 strand at a molar ratio of 1∶1.1 of thiolated to non-thiolated strands respectively. The self-complementary 51 mer thiolated non-damaged single-stranded DNA oligonucleotide 5′-[Thiol-C6]-d(TTTTT-GGATCAGTCACATTCCGAAGTGTTCACTTCGGAATGTGACTGATCC) was allowed to self hybridize to form a hairpin structure referred to as HP. Prior to adsorption, the desired amount of DNA was reduced using Tris(2-carboxyethyl)phosphine in 0.4 M NaH_2_PO_4_, pH 7.4. The DNA samples were then desalted using BioSpin 6 column (BioRad) pre-equilibrated with the buffer used for the DNA adsorption (0.4 M NaH_2_PO_4_, pH 7.4). The final duplex DNA solution was adjusted to the desired concentration and was spotted immediately onto freshly pre-treated gold surfaces.

Prisms were prepared essentially as described by Nogues *et al* [36], briefly, after treatment of the gold layer by immersion in 1 mM 1-mercapto-undecane-tetraethyleneglycol for 30 seconds, 1 µM aliquots of thiolated DNA oligonucleotides were deposed leading to homogenous spots with surface densities of approximately 1.8×10^12^ molecules per cm^2^.

150 µL of three concentrations of APE1 (37, 74, and 147 nM in NIR buffer and 74, 147 and 295 nM in “BER+EDTA” buffer) were injected onto the surface with a flow rate of 25 µL/min at room temperature. Sensorgrams were recorded in a SPRi-Plex apparatus and kinetic constants were obtained by fitting the sensorgram curves with the Bia evaluation software using a 1∶1 dissociation Langmuir model.

### MALDI-TOF Mass Spectrometry Analyses of the NIR Pathway

Mass spectrometry measurements were done as described previously [28]. Typically, 10 pmol of lesion containing oligonucleotide duplexes (in 100 µL) were incubated with the APE1 protein (10 nM) in the “NIR” buffer at 37°C for 17 h. The reaction products were precipitated with 2% lithium perchlorate in acetone, dessalted and then dissolved in water prior subjection to the MALDI-TOF MS measurements. The latter MALDI mass spectra were obtained in negative mode on a time-of-flight Biflex mass spectrometer (Bruker, Wissembourg, France) or in the positive mode on a time-of-flight Axima Performance (Shimadzu, Manchester, UK), both equipped with a 337 nm nitrogen laser and pulsed delay source extraction. The matrix was prepared by dissolving 3-hydroxypicolinic acid in 10 mM ammonium citrate buffer and a small amount of Dowex-50W 50×8−200 cation exchange resin (Sigma). Sample (1 µL) was added to matrix (1 µL) and the resulting solution was made homogeneous by stirring. The resulting sample was placed on the target plate and allowed to dry. Spectra were calibrated using reference oligonucleotides of known masses.

### Cell Culture and Silencing of APE1 Expression

All procedures were performed as described previously [28]. HeLa cells (ATCC #CCL-2, U.S.A.) were routinely grown at 37°C in 5% CO_2_ in Dulbecco minimal essential medium supplemented with 10% foetal calf serum, 2 mM glutamine, 100 U/mL penicillin and 100 mg/mL streptomycin. The siRNAs sequences used to decrease APE1 and NTH1 in HeLa cells have been taken from previously described studies [37]. The siRNA specific to mouse major AP endonuclease, APEX, was used as negative control in both cases. HeLa cells were transfected with the siRNA oligonucleotides using Lipofectamine 2000 (Invitrogen, France) according to the manufacturer’s instructions. Cells were plated at 2×10^6^ cells per Petri dish, incubated for 18 h and then transfected with the specific siRNA. Transfection efficiency was monitored by co-transfection of control cells with pmaxGFP vector (Amaxa, Germany). After 72 h, cells were collected and whole cell-free extracts were prepared as described [21]. Briefly, the cells pellet was washed in ice-cold PBS and incubated for 15 min at 4°C with rocking in the lysis buffer containing 1 M KCl, 80 mM HEPES (pH 7.6), 0.1 mM EDTA, 2 mM DTT, 0.3% NP-40 and protease inhibitor cocktail (Complete EDTA-free, Roche). After centrifugation at 65000 rpm for 1 h at 4°C, the supernatants were collected and stored in 50% glycerol at −20°C for immediate use or at −80°C for longer storage. To measure the protein level the antibodies’ revealed bands in western blots were quantified by densitometry with ImageJ software (National Institutes of Health, Bethesda, MD, USA).

## Results

### Human APE1 Cleaves Oligonucleotide Duplexes Containing Ethenobases

**Figure 2:**
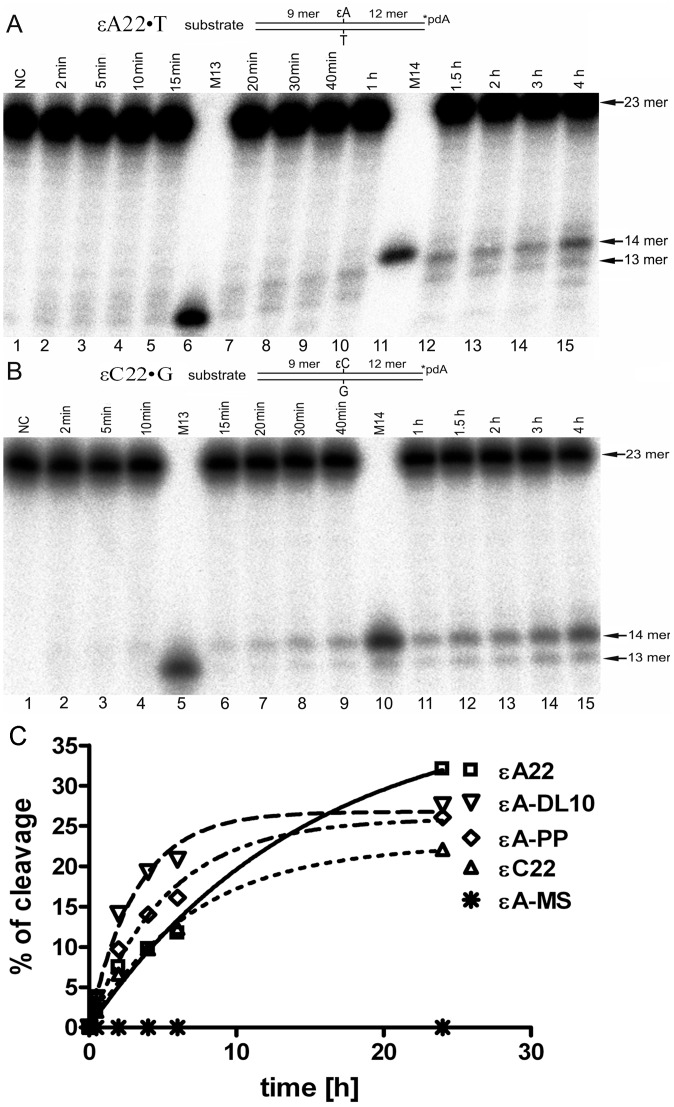
Time dependent cleavage of the oligonucleotide duplexes containing a single ε-base by APE1. A solution of 10 nM of the 3′-[^32^P]-labelled oligonucleotide duplexes was incubated with 5 nM (A,B) or 10 nM (C) APE1 for varying periods of time at 37°C. (**A**) εA22•T duplex. Lane 1, control, non-treated duplex; lanes 2–5, 7–10 and 12–15, as 1 but incubated with APE1 from 2 min to 4 h; lane 6, 13 mer size marker; lane 11, 14 mer size marker. (**B**) εC22•G duplex. Lane 1, control, non-treated duplex; lanes 2–4, 6–9 and 11–15, as 1 but incubated with APE1 from 2 min to 4 h; lane 5, 13 mer size marker; lane 10, 14 mer size marker. The arrows denote the position of the 23-mer, 13-mer and 14-mer fragments, respectively. (**C**) Graphical representation of time dependent kinetics of APE1-catalyzed cleavage activity of various oligonucleotide duplexes containing either single εA or εC residue. A solution of 10 nM of the 3′-[^32^P]-labelled oligonucleotide duplex was incubated with 10 nM APE1 for varying periods of time at 37°C. The amount of cleavage product was quantified and plotted against incubation time. For details see Materials and Methods.

Previously, we have investigated the cleavage of εA•T and εC•G duplexes by *E. coli* Nfo, *S. cerevisiae* Apn1 and human APE1 NIR-AP endonucleases using a limited number of sequence contexts and short incubation times from 5 to 30 min. Here, to examine whether ε-adducts could be substrates for the NIR AP endonucleases we chose 22 mer oligonucleotide duplexes with the sequence context previously used to study the repair of pyrimidine-derived hydantoins [28]. The 3′-[^32^P]-labelled 22 mer εA22•T and εC22•G duplexes were incubated with Nfo, Apn1 and APE1 proteins for extended periods of time and the reaction products were analyzed on a 20% denaturing PAGE. To discriminate between BER and NIR activities we used 3′-[^32^P]-labelled 13 mer and 14 mer synthetic size markers that migrate to the positions corresponding to DNA glycosylase cleavage product (n mer) and AP endonuclease cleavage product (n+1 mer), respectively. As shown in Figure 2A, prolonged incubation (up to 4 h) of εA22•T with APE1 lead to the generation of a cleaved fragment that migrated to a position corresponding to a 14 mer size marker (lanes 6–15) but with a shorter migration than a 13 mer size marker fragment (lane 5). This cleavage pattern indicates that APE1 incised 5′ next to ε-adduct and generated a 14 mer cleavage product containing 5′-dangling εA residue. Similar results were obtained when 3′-[^32^P]-labelled εC22•G duplexes were incubated with APE1 (Figure 2B). As expected, APE1 cleaves εA22•T and εC22•G duplexes only under NIR condition and no detectable activity was observed under BER (Figure S1). Unexpectedly, we did not detect any specific cleavage of 3′-labelled εA22•T and εC22•G when using NIR-AP endonucleases from bacteria, Nfo and yeast, Apn1, furthermore with prolonged incubation times Nfo and Apn1 degraded DNA substrates in a non-specific manner (Figure S2A). Importantly, under the experimental conditions used Nfo and Apn1 exhibited activity towards their classic DNA substrates indicating that the enzymes used were fully proficient (Figure S2B). These results suggest that human APE1 has a broader substrate specificity compared to that of the endonuclease IV family of AP endonucleases, and that this property could be specific to mammalian Xth-family of AP endonucleases.

Next, we examined whether APE1 recognizes εA and εC adducts present in a number of DNA sequence contexts different from that of the 22 mer oligonucleotide used above. For this purpose we constructed nine duplex oligonucleotides designated as C21, DL, DL10, PN, PP, RT and MS in which a single ε-base was placed in the different sequence contexts (Table 1). The εN-C21•N, εN-RT•N, εN-PN•N, εN-DL•N, εN-MS•N, εA-DL10•T, εA-PP•T, and εA28•T duplex oligonucleotides, where εN is either εA or εC, and N is either T or G, were incubated in the presence of APE1 for varying periods of time at 37°C. Analysis of the reaction products showed that, in addition to 22 mer duplex substrate, APE1 cleaved five oligonucleotide duplexes (εN-C21•N, εN-RT•N, εA-DL10•T and εA-PP•T) out of nine tested (Table 1). We did not detect cleavage of εN-PN•N, εN-DL•N, εN-MS•N and εA28•T oligonucleotide duplexes (Table 1). As shown in Figure 2C, APE1 cleaves ε-base containing duplexes with varying efficiency, among those tested, εA-DL10•T being the most preferred substrate. It should be noted that the cleavage activity of APE1 towards εA and εC present in εN22•N, εN-C21•N, εN-RT•N, εN-PN•N, εN-DL•N and εN-MS•N duplexes is rigorously matched for the two substrates. Taken together these data indicate that APE1-catalyzed NIR activity on ε-bases is strongly dependent on sequence context. Analysis of the sequence contexts presented in Table 1 did not reveal any sequence specific motif, although GC rich context strongly inhibits APE1 cleavage on εN-DL•N and εN-MS•N duplexes. Importantly, APE1 can cleave next to an ε-base in both configurations when the lesion is close to the 5′ terminus at position 10 (εA-DL10) and also when it is located at the centre of the oligonucleotide at position 20 (εA-PP) (Figure 2C and Table 1).

### Kinetic Parameters for the Incision of Duplex DNA Containing a Single εA or εC Adduct by APE1

For quantitative evaluation of the substrate specificities of APE1, the kinetic parameters of cleavage of εA22•T and εC22•G duplex oligonucleotides were measured under steady state conditions. As shown in Table 2, the *K*_M_ values of APE1 for both substrates are extremely low 4.3 nM and 2.1 nM for εA and εC respectively, indicating a very high affinity of the human protein for ε-adducts when present in duplex DNA. As expected from previous time-dependent kinetics of incision (Figure 2) the *k*_cat_ values of APE1 for the substrates were very low (≈0.001 min^−1^) indicating extremely slow turnover rate of the enzyme. The *k*_cat_ value means that 1 molecule of APE1 hydrolyses 1 molecule of εA•T substrate in 18.5 hours. The *k*_cat_/*K*_M_ values of APE1 for εA•T and εC•G duplexes (0.2–0.8 µM^−1^•min^−1^) were about 100 folds lower compared to that for αdA•T and DHU•G (16–19 µM^−1^•min^−1^) [26]. Direct extrapolation of these biochemical data to *in vivo* conditions suggest the NIR pathway would remove ε-bases quite inefficiently compared to oxidized bases. Comparison of the *k*_cat_/*K*_M_ values of DNA glycosylases ANPG for εA•T (2.5 µM^−1^•min^−1^) [38] and hTDG for εC•G (0.38 µM^−1^•min^−1^) [14] with that of APE1 for the corresponding DNA substrates revealed that ANPG is 12 times more efficient whereas hTDG is 2 times less efficient than APE1. These results may suggest that NIR may serve as an efficient back-up repair pathway for BER to remove εC. Whereas for the removal of εA residues in DNA NIR ought to considered as a minor pathway.

**Table 2:** Kinetic constants of APE1-catalyzed NIR activity on oligonucleotide duplexes containing single εA or εC residue.

	εA22•T	εC22•G
aK_M_ [nM]	4.3±0.7	2.1±0.95
V_max_ [pM/min]	3.9±1,35	3.6±0.7
k_cat_ [1/min]	0.9×10^−3^	1.7×10^−3^
k_cat_/K_M_	0.21×10^−3^	0.81×10^−3^
bIC_50_ [nM]	5.1±1.1	10.0±1.2

a Kinetic parameters of NIR activity of APE1 towards εA22•T and εC22•G duplexes under NIR reaction conditions. To determine K_M_ and k_cat_, the linear velocity was measured and the constants were calculated using Lineweaver-Burk plots. All determinations were performed at least three times.

b IC_50_ is the concentration of the inhibitor required for 50% inhibition of the AP site cleavage activity of APE1.

Importantly, the APE1-catalyzed incision of duplex DNA containing ε-bases and alpha-anomeric 2′-deoxynucleosides (αdN) is highly sensitive to the Mg^2+^ concentration, ionic strength and pH (Figure S1 and [26]). This implies that APE1 recognizes various DNA base lesions with distinct structures in the same manner. Previously, we have isolated an APE1-D308A mutant carrying a single amino acid substitution and which exhibits dramatically reduced NIR activity on αdA residues but still contains robust AP endonuclease activity [34]. Therefore, we examined whether NIR-deficient APE1-D308A mutant can incise duplex DNA containing ε-bases. As expected, at variance to wild type APE1, the APE1-D308A mutant was not able to cleave εA-DL10•T duplex (Figure S3A). In control experiments, the APE1-D308A mutant displayed good activity on synthetic AP site but failed to incise αdA•T duplex (Figure S3B). These results strongly suggest that the active site amino acid residues involved in the recognition of ε-bases and αdA are the same.

### Affinity of APE1 for Duplex DNA Containing a Single εA or εC Residue

The low *K*_M_ values of APE1 for DNA duplexes containing ε-bases imply that the protein has a high affinity for εA•T and εC•G duplexes and binds strongly to them. Therefore, we examined the interaction of 3′-[^32^P]-labeled εA22•T and εC22•G duplexes with APE1 using an electrophoretic mobility shift assay (EMSA). However, under the experimental condition used, bands on EMSA gel showed smeared pattern of migration that were difficult to quantify reliably (Figure S4). Interestingly, analysis of the migration pattern of APE1 complexes showed that DNA bands were shifted towards higher molecular mass multiprotein complexes suggesting that several molecules of APE1 may bind to a single molecule of duplex DNA (Figure S4).

As an alternative approach for quantitative measurement of APE1-DNA interactions we used Surface Plasmon Resonance imagery (SPRi) technology. For this, thiolated DNA oligonucleotides including regular single-stranded T22 oligonucleotide, A22•T and εA22•T duplexes and unimolecular hairpin oligonucleotide, HP were immobilized on gold surfaces of an SPRi biochip. Varying concentrations of the APE1 protein in either NIR or “BER+EDTA” buffer were flowed across the surface and electrostatic biopolymer adsorption was measured in real-time. Analysis of the resulting SPRi binding profiles revealed that under both NIR and BER conditions APE1 binds with high efficiency to regular single-stranded DNA (Figure S5). Surprisingly, under NIR conditions APE1 does not bind to HP, a regular hairpin structure DNA oligonucleotide, that lacks double-strand break ends, whereas it binds with good efficiency to regular A22•T duplex that has identical sequence context with that of HP (Figure S5). Analysis of binding constants derived from fitting the curves to a 1∶1 Langmuir binding model showed that: (i) APE1 had very low affinity for HP; (ii) under both conditions, *K*_d_ values of APE1 for A22•T and εA22•T duplexes were very similar (Table S1). Taken together these results suggest that APE1 can bind with good affinity to single-stranded DNA regions and double-strand break ends thus rendering difficult direct measurement of K_D_ values for short duplex DNA fragments.

**Figure 3:**
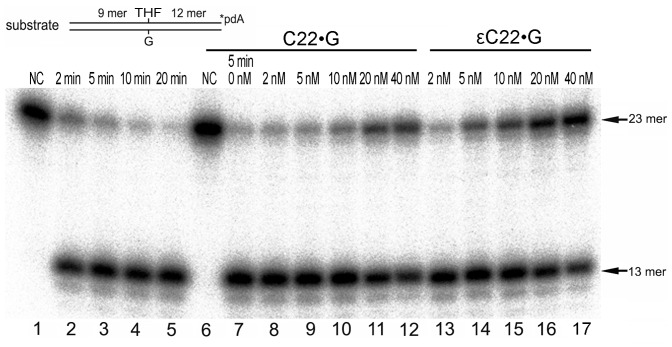
Inhibition of AP site cleavage activity of APE1 by C22•G and εC22•G oligonucleotide duplexes. A solution of 1 nM of 3′-[^32^P]-labelled THF•G oligonucleotide duplex was incubated with 0.5 nM APE1 for 2–20 min at 37°C in the presence of increasing amounts of non-labelled 22 mer C22•G or εC22•G duplexes under NIR conditions. Lane 1, control, non-treated THF•G duplex; lane 2, as 1 but APE1 for 2 min; lane 3, as 2 but 5 min; lane 4, as 2 but 10 min; lane 5, as 2 but 20 min; Lane 6, as 1; lane 7, THF•G duplex with APE1 for 5 min; lane 8, as 7 but 2 nM C•G; lane 9, as 7 but 5 nM C•G; lane 10, as 7 but 10 nM C•G; lane 11, as 7 but 20 nM C•G; lane 12, as 7 but 40 nM C•G; lane 13, as 7 but 2 nM εC22•G; lane 14, as 7 but 5 nM εC22•G; lane 15, as 7 but 10 nM εC22•G; lane 16, as 7 but 20 nM εC22•G; lane 17, as 7 but 40 nM εC22•G. For details see Materials and Methods.

Next, to measure the affinity of APE1 for its DNA substrates we took advantage of a competitive inhibition assay. For this the cleavage rate of 3′-[^32^P]-labelled THF•G or THF•T by APE1 was measured under NIR condition in the presence of increasing concentrations of non-labelled 22 mer εC•G and C•G or εA•T and A•T duplexes, respectively. As shown in Figure 3, the presence of 40 fold molar excess of regular 22 mer C•G duplex inhibited only 30% of AP endonuclease activity (lane 12) whereas the same concentration of εC•G inhibited 70% of AP endonuclease activity (lane 17) suggesting that APE1 has a higher affinity towards εC residue when present in duplex DNA as compared to regular base. Using this approach we measured the effective concentrations of εA•T (EC_50_ = 5.1 nM) and εC•G (EC_50_ = 10.0 nM) duplexes that reduce the rate of APE1-catalyzed AP endonuclease activity by half (Table 2). The EC_50_ values of ε-bases containing DNA are very low and close to K_M_ values thus suggesting that APE1 has a high affinity for ε-bases when present in duplex DNA and most likely forms a specific complex with ε-bases *in vivo*.

### Study of the Mechanism of Action of APE1 and DNA Glycosylases on εA and εC Residues by MALDI-TOF Mass Spectrometry

**Figure 4:**
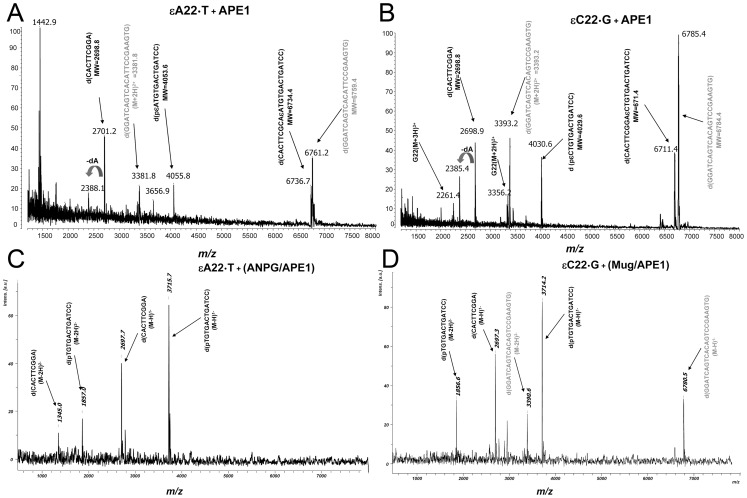
MALDI-TOF MS analysis of the mixture of oligonucleotides arising from the incubation of the 22 mer oligonucleotide duplexes containing a single εA or εC residue with APE1 or DNA glycosylases. Typically, a solution of 10 pmol of the lesion containing oligonucleotide duplexes was incubated with either 10 nM APE1 under NIR conditions at 37°C for 17 h or 50 nM ANPG or 50 nM MUG at 37°C for 30 min and subsequently with 10 nM APE1 at 37°C for 30 min under “BER+Mg^2+^” reaction conditions. (A) Treatment of εA22•T duplex with APE1; (B) Treatment of εC22•G duplex with APE1; (C) Treatment of εA22•T duplex with ANPG and APE1; (D) Treatment of εC22•G duplex with MUG and APE1. Peaks corresponding to complementary strands are indicated in grey. For details see Materials and Methods.

The molecular mechanism of APE1 action on ε-bases was derived from the analysis of the migration pattern of 3′-end labelled cleavage DNA fragments in denaturing PAGE [21]. To further substantiate the mechanism of action of human AP endonuclease acting on ε-bases by a different approach, we characterized the nature of APE1 cleavage products by mass spectrometry. We performed MALDI-TOF MS analyses of the reaction products of APE1 and εN-specific DNA glycosylases when acting on εA22•T and εC22•G duplex oligonucleotides. The mass spectrum of the reaction products resulting from the incision of εA22•T by APE1 showed two mono-charged cleavage product peaks: one at [M+H]^+^ = 2701.2 Da corresponding to the 9 mer oligonucleotide released 5′ upstream to the lesion 5′-CACTTCGGA (with a calculated mass 2698.8 Da), and the other one with molecular mass [M+H]^+^ = 4055.8 Da corresponding to an expected 13 mer APE1-cleavage fragment 5′-p-εATGTGACTGATCC released 3′ downstream to the lesion with a calculated mass 4053.6 Da (Figure 4A). In addition to cleavage products there are mono-charged peaks, [M+H]^+^ = 6761.2 Da corresponding to the complementary strand with a calculated mass 6759.4 Da and the non-cleaved εA-containing strand [M+H]^+^ = 6736.7 Da with a calculated mass 6734.4 Da. Furthermore, after incision APE1 extends the nick to a gap by its 3′→5′ exonuclease activity generating a shorter 8 mer downstream cleavage fragment 5′-CACTTCGG ([M+H]^+^ = 2388.1 Da) (Figure 4A). Analogous results were obtained when analyzing the mass spectrum of the reaction products resulting from the incision of εC22•G by APE1 (Figure 4B). APE1 generates two mono-charged cleavage product peaks: one at [M+H]^+^ = 2698.9 Da corresponding to the 9 mer oligonucleotide released 5′ upstream to the lesion 5′-CACTTCGGA (with calculated mass 2698.8 Da), and the other one with molecular mass [M+H]^+^ = 4030.6 Da corresponding to a 13 mer oligonucleotide 5′-p-εCTGTGACTGATCC (with calculated mass 4029.6 Da) (Figure 4B). These results indicate that APE1 cleaves duplex DNA 5′ next to εA and εC bases thus unambiguously confirming the mechanism of action of human AP endonuclease on ε-bases. Importantly, analysis of the mass spectrum of the cleavage DNA fragments did not reveal any products of the decomposition of ε-bases implying that APE1 cleaves next to the original base adduct and not its degradation products.

In a 2^nd^ experiment, we used MALDI-TOF MS to compare the mechanism of action of two monofunctional DNA glycosylases ANPG and MUG on εA22•T and εC22•G duplexes, respectively (Figure 4C–D). To incise AP sites generated by ANPG and MUG we performed reactions in the presence of APE1 under “BER+Mg^2+^” condition. As expected, analysis of the two mass spectra revealed two mono-charged peaks one at [M–H]^−^ = 2697 Da corresponding to the 9 mer oligonucleotide released 5′ upstream to the lesion 5′-CACTTCGGA (calculated mass 2698.8 Da) and the other one at [M–H]^−^ = 3715 Da corresponding to the 12 mer oligonucleotide released 3′ downstream to the lesion 5′-p-TGTGACTGATCC (calculated mass 3716.4) (Figure 4C–D). In conclusion, these results corroborate with data described above obtained using denaturing PAGE separation technique (Figure 2).

### *In vitro* Reconstitution of the NIR Pathway for εA and εC Residues

**Figure 5:**
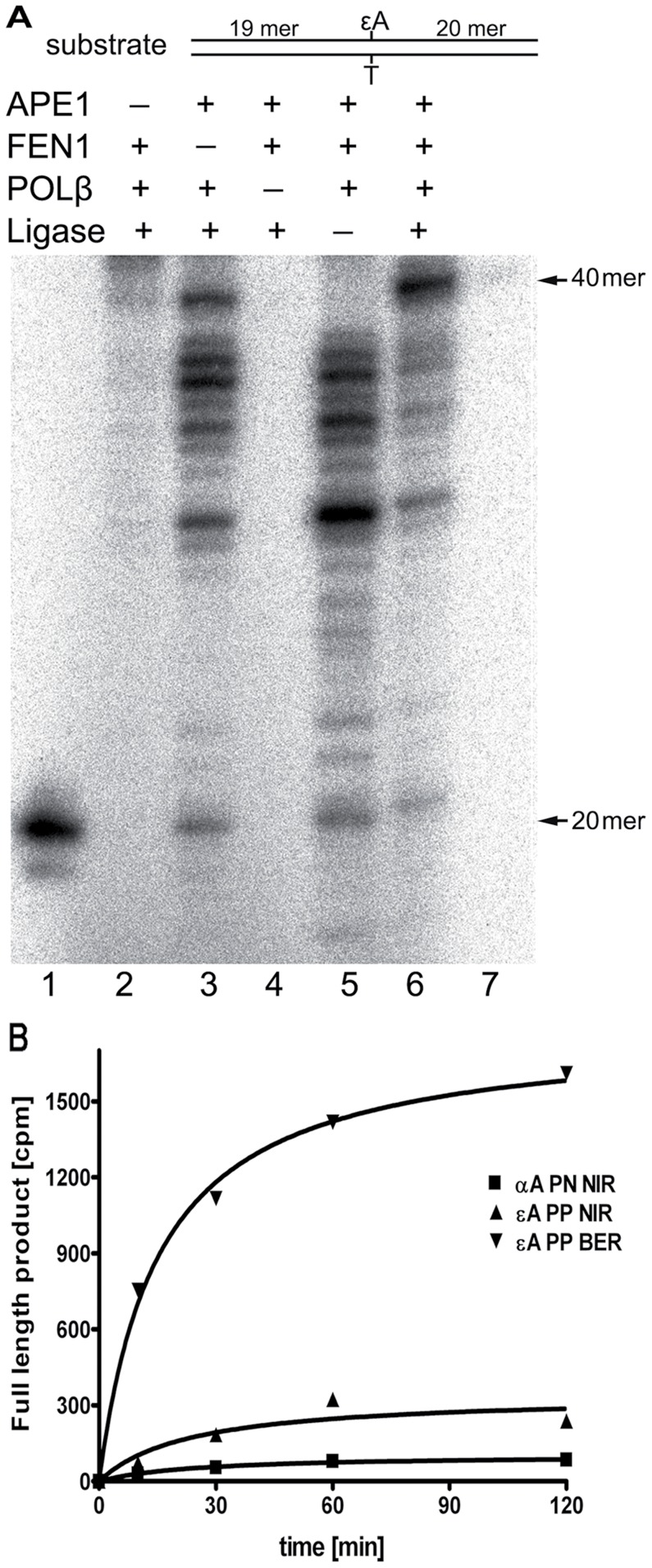
*In vitro* reconstitution of the repair pathways for εA and αdA residues. (**A**) *In vitro* reconstitution of the long-patch NIR pathway using an oligonucleotide duplex containing single εA residue. A solution of 10 nM of non-labelled 40 mer εA-PP•T oligonucleotide duplex was incubated for 3 h at 37°C in the presence of DNA repair proteins. Lane 1, 20 mer size marker; lane 2, εA-PP•T incubated with all proteins except APE1; lane 3, except FEN1; lane 4, except POLβ; lane 5, except ligase; lane 6, in the presence of all proteins; lane 7, 40 mer size marker. (**B**) Time dependent formation of the radioactively labelled full-length product during *in vitro* reconstitution of the BER and NIR pathways initiated by ANPG and APE1, respectively. The 40 mer εA-PP•T and 34 mer αdA•T duplexes were incubated with DNA repair proteins in the presence of [α-^32^P]dATP. At defined time intervals, samples were withdrawn to stop the reaction, then the reaction products were separated by denaturing gel electrophoresis and the amounts of of 40 mer and 34 mer products were measured. Full-length product formation: in the BER pathway reconstitution using 40 mer εA-PP•T (upside-down filled triangle); in the NIR pathway reconstitution using 40 mer εA-PP•T (right-side up filled triangle) and 34 mer αdA•T (filled square). For details see Materials and Methods.

Previously, we have reconstituted *in vitro* the human NIR pathway for the oligonucleotide duplexes containing αdA and hydantoin residues using four purified proteins APE1, flap endonuclease 1 (FEN1), DNA polymerase β (POLβ) and T4 DNA ligase (LIG) [28], [34]. Therefore in the present work, we examined whether extremely slow cleavage rates of the εA•T duplex by APE1 could nevertheless promote removal of ε-bases in a DNA glycosylase-independent manner. As shown in Figure 5A, incubation of a 40 mer duplex oligonucleotide containing a single εA in the presence of all four proteins: APE1, FEN1, POLβ, LIG, [α-^32^P]dATP and non-labelled cold dNTPs for 3 h at 37°C generated labelled 20 mer cleavage fragment, 40 mer full-length product and several intermediate sized DNA fragments (21 to 39 mer) (lane 6). No incorporation of [α-^32^P]dATP was observed in the absence of POLβ (lane 4); though, a small non-specific incorporation of [α-^32^P]dATP in the absence of APE1, possibly due to non-specific synthesis by POLβ, was also detected (Figure 5A, lane 2 and Figure S6). In the absence of either FEN1 or LIG, POLβ could efficiently initiate strand-displacement DNA synthesis and produce intermediate sized DNA fragments (21–39 mer) (Figure 5A, lanes 3 and 5) including full-length 40 mer in the latter case (lane 3). Interestingly, in the presence of FEN1 and the absence of LIG, no full-length 40 mer product was observed (lane 5) suggesting that FEN1 can limit strand-displacement synthesis of POLβ. Also, in the presence of all four proteins, we observed efficient generation of the full-length 40 mer product and a decrease of the yield of intermediate sized DNA fragments (21–39 mer) (lane 6). Together these results suggest that APE1 cleaves 5′ next to εA and generate 19 mer fragment with 3′-OH termini, which are then extended by POLβ to 20 mer and 21–40 mer products by adding one and/or several nucleotides. POLβ catalyzed synthesis generates a flap-structure which is cleaved by FEN1 to remove 5′-dangling εA residues and generate single-strand breaks, which are then sealed by LIG to restore 40 mer DNA fragments. These data indicate that, under the reaction conditions that enable APE1-catalyzed incision of εA•T duplex, DNA polymerase synthesis and ligation, εA residues can be removed in the DNA glycosylase-independent manner resulting in the restoration of the primary DNA sequence. In control experiment, we have reconstituted the repair of a 34 mer of αdA•T duplex in the presence of pure proteins and [α-^32^P]dATP for 1 h at 37°C. As expected, overall the repair of αdA residues was similar to that of εA (Figure S7). Nevertheless, contrary to εA•T repair, when using αdA•T duplex no formation of the full-length 34 mer product was observed in the absence of FEN1 and/or LIG, also few intermediate sized DNA fragments were generated when all DNA repair proteins were present (Figure S7) implying that the APE1-catalyzed slow incision rate of DNA duplexes containing an ε-base may stimulate strand-displacement synthesis by POLβ.

To quantify the relative efficiency of APE1-catalyzed repair of εA we measured the time dependent formation of the radioactively labelled 40 mer full-length product in an *in vitro* reconstitution assay and compared it to the repair of εA by DNA glycosylase (ANPG) - initiated BER pathway and to the APE1-catalyzed repair of αdA (Figure 5B and Figure S6). As shown in Figure 5B, ANPG-catalyzed excision of εA resulted in 10-fold higher amount of the 40 mer full-length product compared to that of APE1-catalyzed cleavage of εA•T duplex indicating that repair of εA residues in the BER pathway is much more efficient compared to the NIR pathway. Interestingly, although APE1 cleaved αdA-containing duplex DNA at much higher rates compared to εA•T duplex, the repair efficiency of the 34 mer αdA•T duplex by APE1 was comparable to that of the 40 mer εA•T duplex (Figure 5B and Figure S6A-B). This apparent inconsistency might be explained by the high proportion of strand-displacement synthesis during εA repair compared to αdA and consequently higher incorporation of [α-^32^P]dATP by POLβ in the 40 mer product (Figure 5A and Figure S7).

### DNA Repair Activities on εA and εC Containing DNA in Human Cell-free Extracts

**Figure 6:**
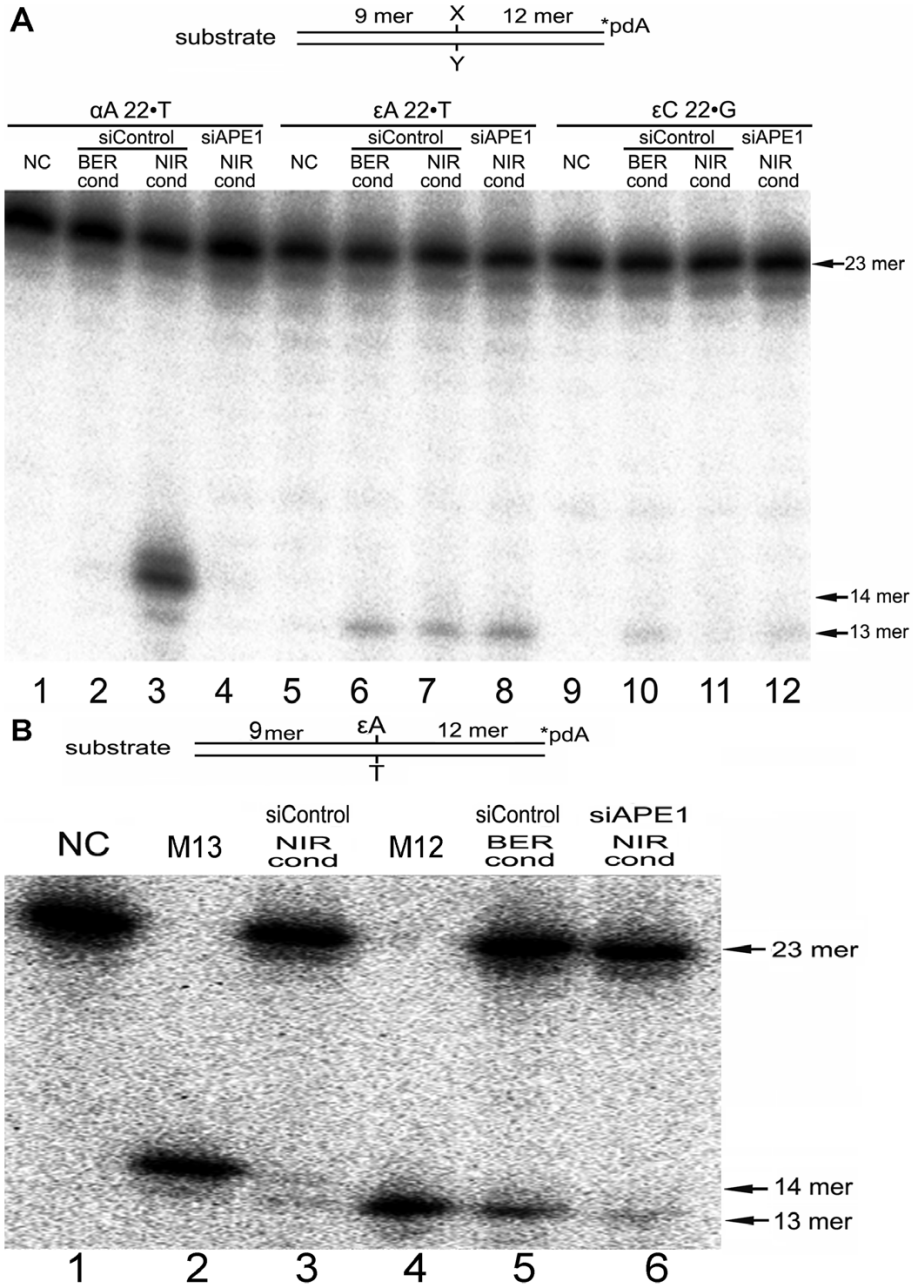
DNA repair activities towards αdA•T, εA22•T and εC22•G oligonucleotide duplexes in HeLa cell extracts. 3′-[^32^P]-labelled oligonucleotide duplexes were incubated in the presence of cell-free extracts from siRNA-treated HeLa cells under either “BER+EDTA” or “NIR+ZnCl_2_” conditions. Cell-free extracts were prepared from HeLa cells treated either with 100 nM of non-specific siRNA (siControl), or with 100 nM of APE1-specific siRNA (siAPE1). (**A**) 10 nM of 3′-[^32^P]-labelled αdA•T, εA22•T and εC22•G oligonucleotide duplexes were incubated for 1 h at 37°C in the presence of 0.5 µg of cell-free extracts. Lane 1, control, non-treated αdA•T duplex; lane 2, as 1 but probed with control extract under BER+EDTA conditions; lane 3, as 2 but under NIR+ZnCl_2_ condition; lane 4, as 1 but probed with APE1-silenced extract under NIR+ZnCl_2_ conditions; Lane 5, control, non-treated εA22•T duplex; lane 6, as 5 but probed with control extract under BER+EDTA conditions; lane 7, as 6 but under NIR+ZnCl_2_ conditions; lane 8, as 5 but probed with APE1-silenced extract under NIR+ZnCl_2_ conditions; Lane 9, control, non-treated εC22•G duplex; lane 10, as 9 but probed with control extract under BER+EDTA conditions; lane 11, as 10 but under NIR+ZnCl_2_ conditions; lane 12, as 9 but probed with APE1-silenced extract under NIR+ZnCl_2_ conditions. (**B**) 10 nM of 3′-[^32^P]-labelled εA22•T oligonucleotide duplexes were incubated for 3 h at 37°C in the presence of 5 µg of cell-free extracts. Lane 1, control, non-treated εA22•T duplex; lane 2, 14 mer size marker; lane 3, as 1 but probed with control extract under NIR+ZnCl_2_ conditions; lane 4, 13 mer size marker; lane 5, as 1 but probed with control extract under under BER+EDTA conditions; lane 6, as 1 but probed with APE1-silenced extract under NIR+ZnCl_2_ condition. For details see Materials and Methods.

Data obtained with the purified APE1 protein demonstrated the redundancy of BER and NIR pathways in the removal of ε-bases when present in duplex DNA. To ascertain the putative role of the NIR pathway *in vivo*, we examined AP endonuclease and DNA glycosylase activities in cell-free extracts from HeLa cells. To distinguish NIR and BER activities in the extracts we used 3′-[^32^P]-labelled εA22•T, εC22•G and αdA•T oligonucleotide duplexes as DNA substrates. When using these substrates DNA glycosylase-mediated base excision generates a 13-mer cleavage fragment, whereas direct cleavage by an AP endonuclease generate a 14-mer fragment. Two buffers “BER+EDTA” to favor DNA glycosylase activities and “NIR+ZnCl_2_” to support APE1-catalyzed NIR function were used to measure DNA repair activities in extracts which were prepared from control and/or APE1-silenced cells as described [26], [27]. In agreement with our previous studies, we observed robust cleavage of αdA•T duplex under NIR conditions (Figure 6A, lane 3) but no activity in the extracts under BER conditions (lane 2) and from siAPE1-treated cells (lane 4) indicating that APE1 is fully functional in cell-free extracts under NIR reaction conditions. As expected, incubation of εA22•T, εC22•G duplexes with extracts under BER conditions generated 13 mer cleavage fragment by DNA glycosylases (lanes 6 and 10). When using 0.5 µg of cell-free extracts and 1 h incubation time at 37°C, no NIR activity on εA22•T and εC22•G duplexes was detected under both BER and NIR conditions (Figure 6A, lanes 6–8 and 10–12). However, as shown in Figure 6B incubation of εA22•T duplex with higher amount of extracts (5 µg) for 3 h resulted in the appearance of a very weak 14 mer cleavage product (lane 3) which was not observed under BER conditions (lane 5) and in the extract from siAPE1-treated cells (lane 6). Importantly, the amounts of 13 mer and 14 mer cleavage products generated by the extracts were similar (lane 3) suggesting that under NIR conditions APE1 contributes significantly to the repair of εA residues. Interestingly, DNA glycosylase activity on εA•T duplex under NIR conditions was inhibited when using 5 µg of cell-free extract (Figure 6B, lanes 3 and 6) compared to when 0.5 µg of extract was used (Figure 6A, lanes 7–8). Nevertheless we did not observe DNA glycosylase inhibition under “BER+EDTA” conditions (Figure 6A, lane 6 *versus* 6B, lane 5) suggesting that the reaction conditions strongly influence protein-DNA interactions in cell-free extracts. We propose that both NIR conditions (pH 6.9 and the presence of 0.1 mM ZnCl_2_) and high cell extract concentrations increase multiple protein binding to DNA and that such non-specific binding could impair the interaction of DNA glycosylase with its DNA substrate. Unexpectedly, we failed to detect APE1-catalyzed cleavage of 22 mer εC•G duplex under similar incubation conditions (data not shown). Again, DNA glycosylase activities were reduced under NIR condition compared to activities in BER buffer (Figure 6A, lanes 7 and 11 *vs* 6 and 10) but stimulated in APE1-silenced extracts compared to activities in control extracts (lanes 7 *vs* 8 and 11 *vs* 12). Taken together, these results suggest that APE1-catalyzed cleavage of εA-containing DNA duplexes may contribute to removal of ε-bases under NIR conditions.

### Activity of APE1 on Oligonucleotide Duplexes Containing Oxidized Base Lesions

**Figure 7:**
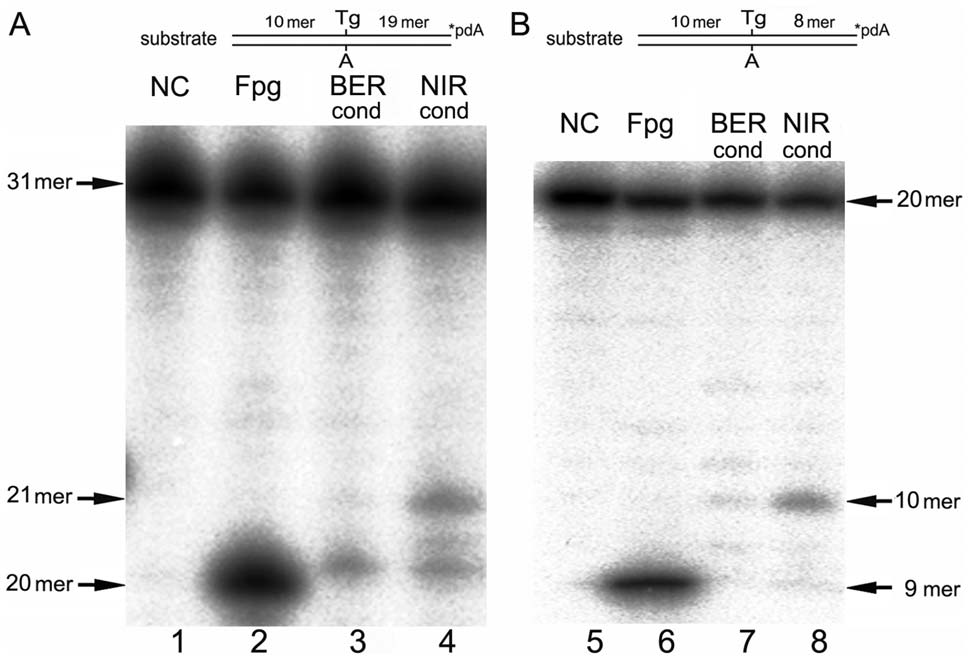
APE1-catalyzed NIR activity towards oligonucleotide duplexes containing single Tg residue. 10 nM of 3′-[^32^P]-labelled 30 mer Tg-RT•A and 19 mer Tg-IW•A oligonucleotide duplexes were incubated for 30 min or 2 h at 37°C in the presence of 20 nM Fpg or 10 nM APE1 under BER+EDTA or NIR conditions, respectively. (**A**) Tg-RT•A and (**B**) Tg19•A duplexes. Lane 1, control non-treated Tg-RT•A duplex; lane 2 as 1 but treated with Fpg under BER condition for 30 min at 37°C; lane 3, as 1 but treated with APE1 for 2 h at 37°C under BER condition; lane 4, as 3 but under NIR condition; lane 5, control non-treated Tg-IW•A duplex; lane 6 as 5 but treated with Fpg under BER condition for 30 min at 37°C; lane 7, as 5 but treated with APE1 under BER condition; lane 8, as 7 but under NIR condition. For details see Materials and Methods.

In the above experiments by the use of prolonged incubation times combined with “optimal” sequence context we have extended the substrate specificity of APE1 to some classical DNA glycosylase substrates such as ε-bases. Next, we examined whether other base damage such as Tg and 8oxoG could also be substrates for APE1 under conditions used in the present study. For this purpose we used 3′-[^32^P]-labelled 19 mer (IW context), 22 mer and 30 mer (RT context) duplex oligonucleotides containing single Tg or 8oxoG residue (Table 1). As a control treatment and also to obtain size markers we incubated DNA substrates with *E. coli* Fpg DNA glycosylase which excises both residues with good efficiency [39], [40]. As shown in Figure 7, both Tg•A duplexes were incised by APE1 under NIR condition generating 10 mer and 21 mer (*n+1* mer) cleavage products (lanes 4 and 8) whereas Fpg generated 9 mer and 20 mer (*n* mer) cleavage products that migrated faster than APE1-generated products (lanes 2 and 6). As expected, under BER condition APE1-catalyzed cleavage of Tg•A duplexes was dramatically reduced (lanes 3 and 7) further confirming that activity on Tg residues is a NIR function of APE1. No cleavage of 22 mer and 30 mer 8oxoG•C duplexes by APE1 was detected under the experimental conditions used (Table 1) suggesting that despite extremely broad substrate specificity, APE1’s DNA damage recognition potential is limited to oxidized purines.

## Discussion

Oxidized bases and exocyclic adducts are the major endogenous DNA lesions that accumulate during aging [41]. LPO generated reactive aldehydes interact with DNA resulting in a number of ε-bases that are highly mutagenic *in vitro* and *in vivo*. Cells evolved several repair mechanisms to remove endogenous DNA base damage from the genome including DNA glycosylase-dependent BER, direct damage reversal and NIR pathways. APE1 is a multifunctional repair enzyme involved in both BER and NIR pathways, redox regulation of the transcription factors and other biological functions such as parathyroid hormone gene regulation and nitric oxide production [42], [43]. Suppression of the APE1 results in embryonic lethality in mice [44], [45] and also inhibits human cell proliferation and/or induces apoptosis [42], [46], indicating that either some or all of the APE1 functions are essential for cell viability. Importantly, reduced APE1 levels increase cellular sensitivity to hydrogen peroxide, menadione, paraquat and ionizing radiation, but not to UV irradiation [47], [48]. Furthermore, spontaneous mutation frequencies in somatic tissues and spermatogenic cells of APEX1 heterozygous knockout mice are increased by two fold compared to wild type mice [49]. These observations strongly support the physiological significance of the DNA repair functions of APE1 in maintenance of genome stability *in vivo*. Previously, we established that the APE1-initiated NIR pathway removes distinct types of oxidative DNA base damage including those that are not processed by DNA glycosylases [26], [27], [28]. Recently, it has been shown that APE1 is able to incise next to bulky 6–4 photoproducts (6-4PPs) generated by UV irradiation in duplex DNA [50]. The ability of APE1 to recognize an extremely large variety of different DNA substrates prompted us to further investigate its substrate specificity towards well known endogenous DNA lesions repaired by DNA glycosylases.

In the present study, we investigated whether the AP endonucleases involved in the NIR pathway recognize exocyclic DNA adducts and other oxidized bases in duplex DNA (Figure 1). The results show, for the first time, that human AP endonuclease, APE1 can incise, in a DNA glycosylase-independent manner, duplex DNA containing εA, εC and Tg residues (Figures 2 and 7). However, APE1-catalyzed cleavage of duplex DNA containing εA and εC residues is strongly dependent on sequence context (Table 1). It should be stressed that the affinity of APE1 to ε-bases and αdA is strongly dependent on the reaction conditions and that mutation of the critical amino acid residue D308 results in complete loss of NIR activity on εA•T, εC•G and αdA•T duplexes (Figures S1 and S3). Unexpectedly, despite common reaction mechanisms and substrate specificity with APE1, the *E. coli* Nfo and *S. cerevisiae* Apn1 NIR-AP endonucleases were not able to cleave ε-base containing duplex oligonucleotides, under the experimental conditions used (Figure S2). It should be noted that Nfo and Apn1 belong to the endonuclease IV family enzymes whereas APE1 belongs to structurally different Xth-family of AP endonucleases [51]. We suggest that this unique versatile substrate specificity of the human AP endonuclease together with its redox-function, which is absent in the homologous *E. coli* Xth protein, were acquired during the evolution of higher eukaryotes. Analysis of kinetic parameters revealed that APE1 cleaves εA•T and εC•G duplexes at extremely slow rates (k_cat_≈0.001 min^−1^) (Table 2) compared to AP sites (15 min^−1^) and αdA (0.31 min^−1^) [34]. Overall, the kinetic parameters of APE1-catalyzed cleavage are quite inefficient as compared to that of the DNA glycosylases implying that in human cells the majority of ε-bases would be removed in the BER pathway (Table 2).

Interestingly, the low *K*_M_ values of APE1 when acting on εA and εC residues indicate high affinity of the human protein to duplex DNA containing ε-bases. To further characterize the APE1-binding affinity we attempted to measure K_D_ values of APE1 for εA22•T and εC22•G duplexes using EMSA. Electrophoretic separation of the complexes of APE1 with duplex oligonucleotides resulted in a smear rather than in discrete shifted bands thus rendering difficult the calculation of the relative intensity of shifted DNA bands (Figure S4). We have also used SPRi technology to measure the affinity of APE1 for DNA substrates. Analysis of SPRi binding curves show that APE1 bound with high efficiency to regular single-stranded DNA but failed to interact with hairpin DNA (Figure S5). Furthermore, APE1 showed similar binding affinity to both regular duplex DNA and to damaged DNA containing εA residues (Table S1). These results suggest a complex mode of APE1 binding to DNA in which proteins bind to several sites in the DNA including base lesions, single-stranded regions and double-strand break ends thus making it very difficult to directly measure K_D_ values for short duplex DNA fragments. Finally, to quantitatively measure APE1 affinity for damaged DNA we used an alternative approach based on a competitive inhibition assay. We demonstrated that ε-base containing duplex oligonucleotides more effectively inhibited AP site cleavage activity of APE1, compared to regular DNA duplexes (Figure 3 and Table 2).

To study the molecular mechanism of action of APE1 on ε-bases we analyzed the AP endonuclease-generated cleavage fragments by MALDI-TOF mass spectrometry. As expected, mass spectrometry measurements confirmed those obtained by denaturing PAGE separation: (*i*) APE1 incised the oligonucleotide duplexes 5′ next to εA and εC residues generating 3′ downstream cleavage fragments that still contain 5′-terminal damaged nucleotide; (*ii*) APE1 degraded 5′ upstream cleavage fragments by its non-specific 3′→5′ exonuclease activity (Figure 4A–B). Previously, it was demonstrated, that ε-bases spontaneously undergo base loss and/or rearrangements to ring-opened forms under physiological conditions [52], [53], [54]. We have found that a ring-opened derivative of εA is the substrate for oxidative damage-specific DNA glycosylases [53] and NIR AP endonucleases (*unpublished observation*). Importantly, the mass spectrum analysis did not reveal any chemical transformations of εA and εC residues in the cleaved fragments suggesting that APE1 recognizes intact ε-base and does not degrade 5′-dangling modified base after phosphodiester bond cleavage. Based on the new substrate specificities of APE1 we performed a complete *in vitro* reconstitution of the human NIR pathway for εA•T oligonucleotide duplex using purified proteins. Incubation of the εA-PP•T duplex in the presence of APE1, FEN1, POLβ and LIG generated a full-length 40 mer repair product (Figure 5A). This result demonstrates that ε-bases in duplex DNA, despite slow incision rates, can be processed in a DNA glycosylase-independent manner *via* the APE1-catalyzed NIR pathway. However, it should be noted that the DNA glycosylase-initiated BER removes εA residues with 10 fold higher efficiency compared to that of APE1-catalyzed NIR (Figure 5B and Figure S6).

Data obtained with the purified proteins support a possible physiological role of the APE1-catalyzed removal of ε-bases. To further examine the roles of different DNA repair pathways to process ε-bases, we measured AP endonuclease and DNA glycosylase activities in cell-free extracts from human cells. To decrease non-specific nuclease activities in the extracts ZnCl_2_ was used instead of MgCl_2_ [26]. It should be stressed that zinc is the second most abundant transition metal in the body after iron [55] therefore it could play a biological role in stimulating APE1-NIR activity *in vivo*. When using higher amounts of extracts and longer incubation times we detected a weak NIR activity on the εA•T oligonucleotide duplex but not on the εC•G duplex (Figure 6B). As expected, BER conditions and APE1-silencing lead to strong inhibition of APE1-catalyzed NIR activity on εA•T. The absence of NIR activity on εC•G duplex may be explained by the presence of endogenous ANPG protein in human cell extracts which could bind to εC residues and inhibit their repair by other DNA repair proteins [19], [56]. Interestingly, APE1-silencing stimulated the DNA glycosylase activities towards ε-bases under NIR conditions (Figure 6A). Analogous stimulation has been observed on pyrimidine-derived hydantoins in the extracts from APE1-silenced cells in our previous work [28] suggesting that either APE1 may inhibit DNA glycosylases by binding to their DNA substrates or that the transcription silencing of APE1 gene induces the expression of DNA glycosylases in HeLa cells. It should be noted that, in contrast to the results obtained with extracts, when using purified ANPG and hTDG, we observed stimulation of DNA glycosylase activities in the presence of APE1 (data not shown) which is in agreement with previous studies [57], [58]. Altogether, these results suggest that in the extracts both APE1 and DNA glycosylases can remove εA residues in DNA.

In addition to ε-bases, APE1 also cleaves Tg-containing DNA although at very slow rates (Figure 7). In mammalian cells Nth1 and Neil1 DNA glycosylases are the main enzymes that remove Tg residues from DNA in cell-free extracts [59], [60]. Quite unexpectedly, double-knockout mice lacking mNth1 and mNeil1 do not accumulate Tg residues in their genomic DNA suggesting the existence of alternative pathways for the removal of Tg *in vivo* [61]. Indeed, it was shown that *in vitro* the nucleotide excision repair (NER) pathway can remove Tg in DNA although with lower efficiency compared to Nth1 and Neil1 [62]. Therefore, we propose that APE1-catalyzed cleavage 5′ next to Tg residue may serve as a back-up pathway together with NER in the absence of DNA glycosylases.

APE1 recognizes diverse types of DNA base lesions including 5,6-dihydropyrimidines, alpha-anomeric nucleotides [26], 5-hydroxypyrimidines [27], formamidopyrimidines [63], ε-bases, thymine glycol (present work Figures 2 and 7) and bulky lesions such as 6–4 photoproduct [50] and benzene-derived DNA adducts [64]. Although APE1’s substrate specificity significantly overlaps with that of DNA glycoyslases, the mechanism of action of a DNA glycosylase is fundamentally different from that of an AP endonuclease. To access the C1’ atom, DNA glycosylases flip-out the abnormal base into a specific pocket, whereas AP endonucleases target the phosphodiester bond in DNA and access it by kinking of the helix [10]. Interestingly, co-crystal structures of APE1 bound to abasic DNA show that the enzyme kinks the DNA helix and binds a flipped-out AP site in a pocket that excludes DNA bases [65]. This raises the question of how APE1 can accommodate various modified bases in its active site? The fact that the chemical structures of APE1’s DNA substrates have very little in common implies that APE1 recognizes damage-induced structural distortions of the DNA helix rather than a modified base itself. This property is somewhat similar to the DNA damage recognition mechanism in the NER pathway that removes a very broad spectrum of bulky, helix-distorting base lesions [66]. Based on these observations we suggest that APE1-catalyzed NIR activity correlates with increased binding to DNA helix distortions and subsequent enzyme-induced drastic bending of a DNA duplex. Importantly, the observation of high affinity binding of APE1 to DNA duplexes containing ε-bases and very slow cleavage rates opens the possibility to co-crystallize the enzyme/substrate complex and resolve its structure. Consequently further structural studies are needed to understand the molecular mechanisms of DNA damage recognition by APE1.

Previous studies have established a major role of the DNA glycosylase-mediated BER pathway in the removal of εA and εC residues in the genome [67]. Paradoxically, *APNG^−/−^* mice deficient for εA-DNA glycosylase activity is not susceptible to vinyl carbamate, an agent which specifically induces εA residues in DNA [15]. Subsequent studies have established the role of mammalian AlkB homologues ABH2 and ABH3 iron-dependent dioxygenases in the repair of εA and εC residues *via* direct DNA damage reversal [18], [19]. However, endogenous levels of εA residues in mouse liver is dependent on APNG status but not on ABH2 one [18]. Furthermore, in contrast to *E. coli* DNA repair deficient cells, single knockout *APNG^−/−^* and *ABH2^−/−^* mice embryonic fibroblasts are not sensitive to chloroacetaldehyde treatment which generates ε-bases in DNA suggesting the existence of a second alternative repair pathway [18]. Biochemical data obtained in the present study point to a possible repair of εA and εC residues by APE1-initiated NIR. Indeed, we have been able to detect very weak APE1-catalyzed cleavage of εA22•T duplex in human cell-free extracts (Figure 6B) suggesting that NIR may function as a minor back-up repair pathway to remove ε-bases from DNA in addition to BER and direct damage reversal pathways. Finally, we propose that although the APE1-catalyzed incision of DNA duplex 5′ next to ε-bases and Tg is very slow, it may play a role in protecting cells against oxidative stress and also in conferring drug resistance to cancer cells. Future studies on NIR-deficient mammalian cell lines are needed to understand the biological roles of APE1-catalyzed DNA repair activities.

## Supporting Information

Figure S1 **Dependence of APE1-catalyzed NIR activity on reaction conditions.** A solution of 10 nM of 3′-[^32^P]-labelled oligonucleotide duplex containing a single ε-base was incubated for 1–2 h at 37°C with 5 nM APE1 under NIR and BER conditions. (**A**) εC22•G and (**B**) εA22•T duplexes. Lanes 1 and 9, control, non-treated duplex; lanes 2 and 10, duplex incubated with 20 nM MUG and ANPG for 20 min and then with 10 nM APE1 for 20 min under “BER+Mg^2+^” reaction condition; lanes 3 and 11, 13 mer size marker; lanes 4 and 14, 14 mer size marker; lanes 5 and 12, duplexes incubated with APE1 for 1 h under NIR conditions; lanes 6 and 13, duplexes incubated with APE1 for 1 h under BER conditions; lanes 7 and 15, duplexes incubated with APE1 for 2 h under NIR conditions; lanes 8 and 16, duplexes incubated with APE1 for 2 h under BER conditions. For details see Materials and Methods.
(TIF)

Figure S2 **Action of various NIR AP endonucleases towards oligonucleotide duplexes containing a single ε-base.** A solution of 10 nM of 22 mer 3′-[^32^P]-labelled εA22•T, εC22•G αdA-RT•T and Tg-RT•A oligonucleotide duplexes was incubated with either Nfo, or Apn,1 or 10 nM APE1 for 30 min and 2 h at 37°C. (**A**) Lane 1, control, non-treated εA22•T; lane 2, as 1 but 20 nM ANPG and 10 nM APE1 under BER+Mg^2+^ conditions; lane 3, 13 mer size marker; lane 4, as 1 but 10 nM Nfo for 30 min; lane 5, as 4 but 2 h; lane 6, as 1 but 10 nM Apn1 for 30 min; lane 7, as 6 but 2 h; lane 8, as 1 but APE1 for 30 min; lane 9, 14 mer size marker; lane 10, as 8 but 2 h; lane 11, control, non-treated εC22•G; lane 12, as 1 but 20 nM MUG and 10 nM APE1 under BER+Mg^2+^ conditions; lane 13, as 11 but 10 nM Nfo for 30 min; lane 14, as 13 but 2 h; lane 15, as 11 but 10 nM Apn1 for 30 min; lane 16, as 15 but 2 h; lane 17, as 11 but APE1 for 30 min; lane 18, as 17 but 2 h. (**B**) Lane 1, control, non-treated αdA-RT•T; lane 2, as 1 but 1 nM Nfo for 30 min; lane 3, as 1 but 1 nM Apn1 for 30 min, lane 4, control, non-treated Tg RT•A; lane 5, as 4 but 20 nM Fpg under BER+EDTA conditions; lane 6, as 4 but 1 nM Nfo for 2 h, lane 7, as 4 but 5 nM Nfo for 2 h; lane 8, as 4 but 1 nM Apn1 for 2 h; lane 9, as 4 but 5 nM Apn1 for 2 h, line 10, control, non-treated εA22•T; lane 11, 13 mer size marker; lane 12, as 10 but 1 nM Nfo for 2 h; lane 13, as 10 but 1 nM Apn1 for 2 h, lane 14, 14 mer size marker, line 15, control, non-treated εC22•T; line 16, as 15 but 1 nM Nfo for 2 h; lane 17, as 15 but 1 nM Apn1 for 2 h. For details see Materials and Methods.
(TIF)

Figure S3 **Comparison of NIR and AP endonuclease activities of APE1-WT and mutant APE1-D308A proteins.** A solution of 10 nM of 22 mer 3′-[^32^P]-labelled εA22•T, THF•T and αdA•T oligonucleotide duplexes were incubated with varying amounts of the APE1 proteins under NIR conditions, and products of the reaction were analyzed using denaturing PAGE. (**A**) εA22•T duplex was incubated with varying amounts of the APE1 WT and APE1-D308A mutant proteins for 4 h at 37°C. Lane 1, control, non-treated εA22•T; lane 2, 20 nM ANPG and 10 nM APE1 under BER+Mg^2+^ conditions; lane 3, 5 nM of APE1-WT; lane 4, 25 nM of APE1-WT; lane 5, 100 nM of APE1-WT; lane 6, 5 nM of APE1-D308A; lane 7, 25 nM of APE1-D308A; lane 8, 100 nM of APE1-D308A. (**B**) THF•T and αdA•T duplexes were incubated with varying amounts of the APE1-WT and APE1-D308A mutant proteins for 5 min at 37°C. Lane 1, control, non-treated THF•T; lane 2, 0.02 nM of APE1-WT; lane 3, 0.1 nM of APE1-WT; lane 4, 0.5 nM of APE1-WT; lane 5, 0.02 nM of APE1-D308A; lane 6, 0.1 nM of APE1-D308A; lane 7, 0.5 nM of APE1-D308A; Lane 8, control, non-treated αdA•T; lane 9, 0.1 nM of APE1-WT; lane 10, 0.5 nM of APE1-WT; lane 11, 2.5 nM of APE1-WT; lane 12, 0.1 nM of APE1-D308A; lane 13, 0.5 nM of APE1-D308A; lane 14, 2.5 nM of APE1-D308A. For details see Materials and Methods.
(TIF)

Figure S4 **Electrophoretic Mobility Shift Assay (EMSA) for binding of APE1 to oligonucleotide duplexes containing a single ε-base.** The standard binding reaction mixture (20 µl) contained 20 mM Hepes-KOH, pH 7.6, 50 mM KCl, 10 µM or 100 µM MgCl_2_, 10 nM of 22 mer 3′-[^32^P]-labelled εA22•T, A22•T, εC22•G or C22•G and 250 nM or 500 nM APE1. The mixture was incubated for 10 min on ice, after which an aliquot was analyzed by electrophoresis on a 8% non-denaturing polyacrylamide gel (19∶1 acrylamide/bisacrylamide) at 160V for 14 h at +4°C. Lane 1, control, εA22•T in 10 µM MgCl_2_; lane 2, as 1 but 250 nM APE1; lane 3, as 1 but 500 nM APE1; lane 4, control, εA22•T in 100 µM MgCl_2_; lane 5, as 4 but 250 nM APE1; lane 6, as 4 but 500 nM APE1; lane 7, control, A22•T in 100 µM MgCl_2_; lane 8, as 7 but 500 nM APE1; lane 9, control, εC22•T in 10 µM MgCl_2_; lane 10, as 9 but 250 nM APE1; lane 11, as 9 but 500 nM APE1; lane 12, control, εC22•T in 100 µM MgCl_2_; lane 13, as 12 but 250 nM APE1; lane 14. as 12 but 500 nM APE1; lane 15, control, C22•G in 100 µM MgCl_2_; lane 16, as 15 but 500 nM APE1. For details see Materials and Methods.
(TIF)

Figure S5 **SPRi kinetic curves of APE1 (37–296 nM) interacting with immobilized DNA on the pre-treated surface.** (**A–C**) Measurements were performed in “BER+EDTA” buffer. (**D–F**) Measurements were performed in NIR buffer. The curves representing the interactions of APE1 with Hairpin DNA (HP) are in blue, regular single-stranded T22 oligonucleotides are in grey, εA22•T duplexes are in green, THF-22•T duplexes in pink and A22•T duplexes in red. For details see Materials and Methods.
(TIF)

Figure S6 **Time kinetics of** ***in vitro*** **reconstitution of the NIR and BER pathways using oligonucleotide duplex containing single αdA or εA residue.** (A,B) A solution of 10 nM of non-labelled oligonucleotide duplexes was incubated at 37°C for various times up to 120 min in the presence of 10 nM APE1, 2 nM FEN1, 0.01 U POLβ and 4 U T4 DNA ligase in a reaction buffer containing 50 mM HEPES-KOH (pH 7.2), 30 mM NaCl, 3 mM MgCl_2_, 2 mM ATP, 0.1 mg/ml BSA, 2 mM DTT, 5 mCi of [α-^32^P]dATP and 50 µM each of dGTP, dCTP and TTP. (A) 34 mer αdA-PN•T duplex. Lane 1, 34 mer size marker; lane 2, 0 min of incubation; lane 3, 10 min; lane 4, 30 min; lane 5, 60 min, lane 6, 120 min; (B) 40 mer εA-PP•T duplex. Lane 1, 0 min of incubation; lane 2, 10 min; lane 3, 30 min; lane 4, 60 min; lane 5, 120 min; lane 6, 20 mer size marker; lane 7, 40 mer size marker. (C) 10 nM of non-labelled 40 mer εA-PP•T oligonucleotide duplex was incubated at 37°C for various times up to 120 min in the presence of 40 nM ANPG, 5 nM APE1, 2 nM FEN1, 0.01 U POLβ and 4 U T4 DNA ligase in the reaction buffer containing 20 mM HEPES-KOH (pH 7.6), 50 mM KCl, 5 mM MgCl_2_, 2 mM ATP, 0.1 mg/ml BSA, 1 mM DTT and 5 mCi of [α-^32^P]dATP. Lane 1, 40 mer size marker; lane 2, 0.5 min of incubation; lane 3, 10 min; lane 4, 30 min; lane 5, 60 min; lane 6, 120 min; lane 7, 20 mer size marker. For details see Materials and Methods.
(TIF)

Figure S7 ***In vitro*** **reconstitution of the long-patch NIR pathway using oligonucleotide duplex containing single αdA residue.** 10 nM of non-labelled 34 mer αA-PN•T oligonucleotide duplex was incubated for 3 h at 37°C in the presence of 10 nM APE1, 2 nM FEN1, 0.02 U POLβ and 20 U T4 DNA ligase in the reaction buffer containing 50 mM HEPES-KOH (pH 7.2), 30 mM NaCl, 3 mM MgCl_2_, 2 mM ATP, 0.1 mg/ml BSA, 2 mM DTT, 5 mCi of [α-^32^P]dATP and 50 µM each of dGTP, dCTP and TTP. Lane 1, αA-PN•T incubated with all proteins except APE1; lane 2, except FEN1; lane 3, except POLβ; lane 4, except ligase; lane 5, in the presence of all proteins. The arrows denote the position of 16 mer cleavage product and 34 mer full-length product. For details see Materials and Methods.
(TIF)

Table S1 **Kinetic constants for APE1-DNA substrate interactions deduced from sensorgrams using Bia evaluation software.**
(DOC)
