# Correction: Highly Mutagenic Exocyclic DNA Adducts Are Substrates for the Human Nucleotide Incision Repair Pathway

**DOI:** 10.1371/annotation/861eeca8-8296-46b7-8bdf-947a0c8db4fa

**Published:** 2013-07-31

**Authors:** Paulina Prorok, Christine Saint-Pierre, Didier Gasparutto, Olga S. Fedorova, Alexander A. Ishchenko, Hervé Leh, Malcolm Buckle, Barbara Tudek, Murat Saparbaev

 Information was missing from the Funding statement. The second sentence of Funding should read, "Russian Federal Program 'Scientific and education personnel for innovative Russia' for 2009-2013 (http://mon.gov.ru) [Nos. 8092 and 8473 to A.A.I. and O.S.F.]." 

