# Supplementary figures and images for: Highly Mutagenic Exocyclic DNA Adducts Are Substrates for the Human Nucleotide Incision Repair Pathway
Source: PLoS One. 2012 Dec 14;7(12):e51776. doi: 10.1371/journal.pone.0051776 (PMC3522590; doi:10.1371/journal.pone.0051776)

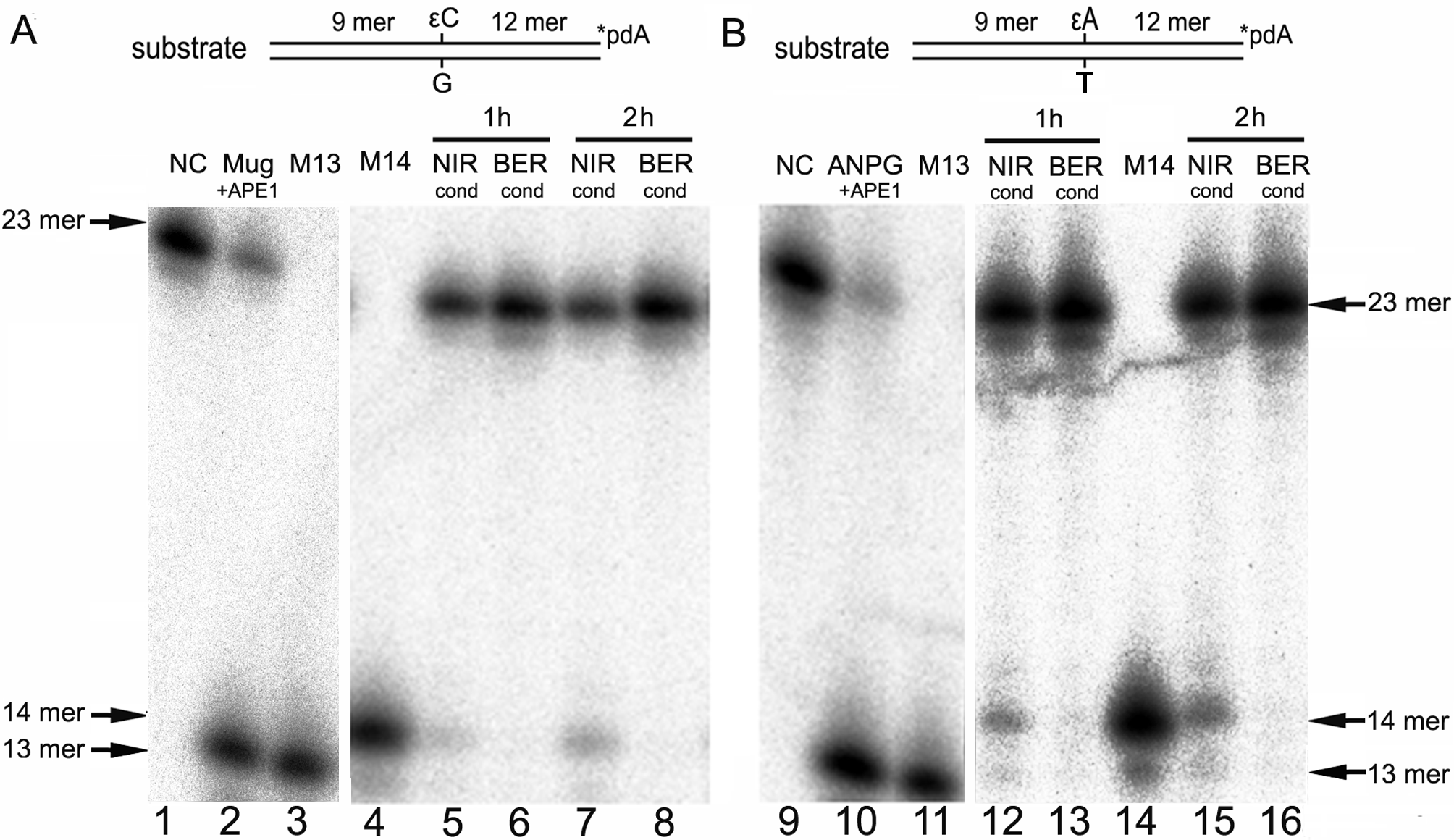

Supplement: Figure S1 — Dependence of APE1-catalyzed NIR activity on reaction conditions. A solution of 10 nM of 3′-[32P]-labelled oligonucleotide duplex containing a single ε-base was incubated for 1–2 h at 37°C with 5 nM APE1 under NIR and BER conditions. (A) εC22•G and (B) εA22•T duplexes. Lanes 1 and 9, control, non-treated duplex; lanes 2 and 10, duplex incubated with 20 nM MUG and ANPG for 20 min and then with 10 nM APE1 for 20 min under “BER+Mg2+” reaction condition; lanes 3 and 11, 13 mer size marker; lanes 4 and 14, 14 mer size marker; lanes 5 and 12, duplexes incubated with APE1 for 1 h under NIR conditions; lanes 6 and 13, duplexes incubated with APE1 for 1 h under BER conditions; lanes 7 and 15, duplexes incubated with APE1 for 2 h under NIR conditions; lanes 8 and 16, duplexes incubated with APE1 for 2 h under BER conditions. For details see Materials and Methods. (TIF) [file pone.0051776.s001.tif]

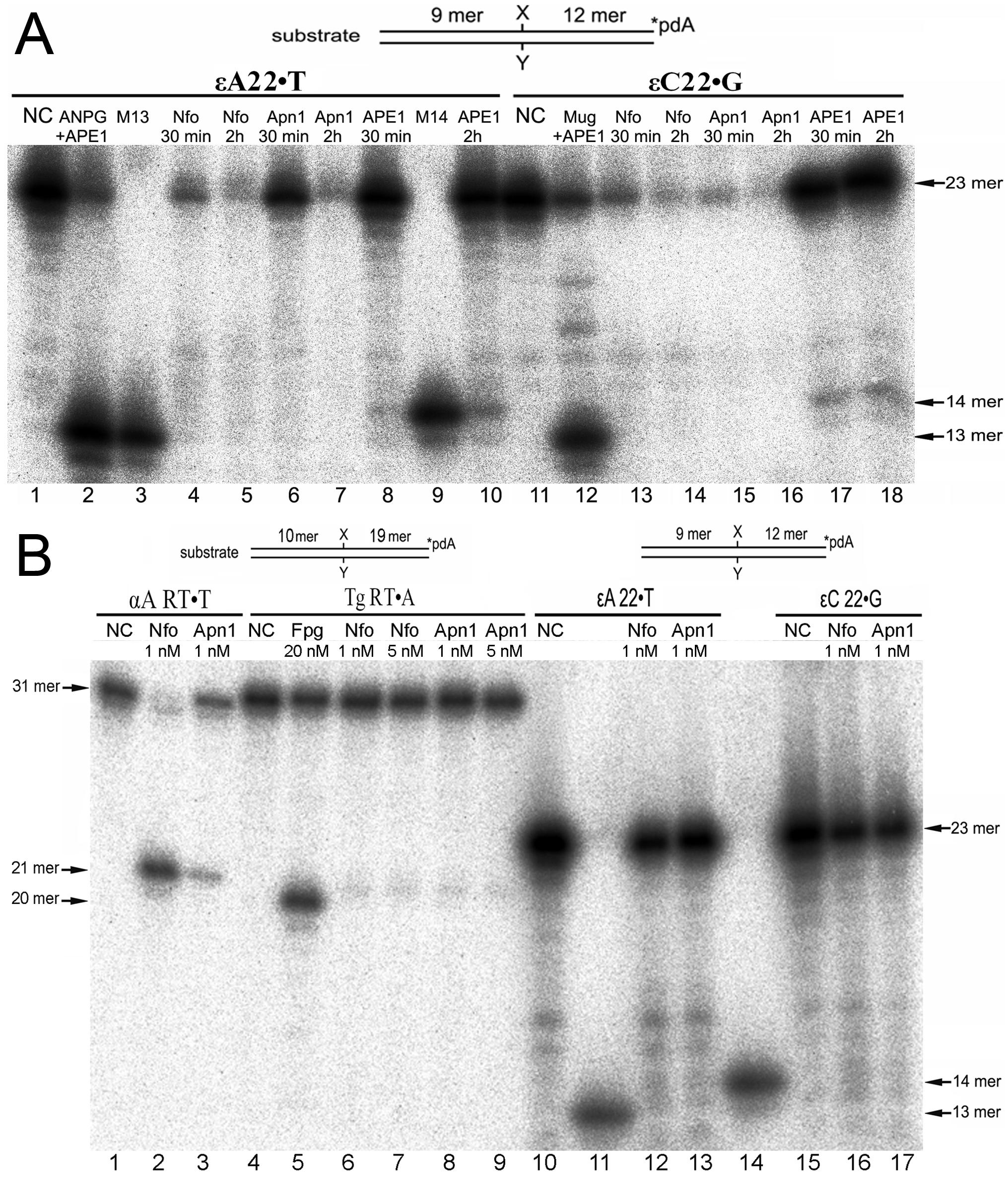

Supplement: Figure S2 — Action of various NIR AP endonucleases towards oligonucleotide duplexes containing a single ε-base. A solution of 10 nM of 22 mer 3′-[32P]-labelled εA22•T, εC22•G αdA-RT•T and Tg-RT•A oligonucleotide duplexes was incubated with either Nfo, or Apn,1 or 10 nM APE1 for 30 min and 2 h at 37°C. (A) Lane 1, control, non-treated εA22•T; lane 2, as 1 but 20 nM ANPG and 10 nM APE1 under BER+Mg2+ conditions; lane 3, 13 mer size marker; lane 4, as 1 but 10 nM Nfo for 30 min; lane 5, as 4 but 2 h; lane 6, as 1 but 10 nM Apn1 for 30 min; lane 7, as 6 but 2 h; lane 8, as 1 but APE1 for 30 min; lane 9, 14 mer size marker; lane 10, as 8 but 2 h; lane 11, control, non-treated εC22•G; lane 12, as 1 but 20 nM MUG and 10 nM APE1 under BER+Mg2+ conditions; lane 13, as 11 but 10 nM Nfo for 30 min; lane 14, as 13 but 2 h; lane 15, as 11 but 10 nM Apn1 for 30 min; lane 16, as 15 but 2 h; lane 17, as 11 but APE1 for 30 min; lane 18, as 17 but 2 h. (B) Lane 1, control, non-treated αdA-RT•T; lane 2, as 1 but 1 nM Nfo for 30 min; lane 3, as 1 but 1 nM Apn1 for 30 min, lane 4, control, non-treated Tg RT•A; lane 5, as 4 but 20 nM Fpg under BER+EDTA conditions; lane 6, as 4 but 1 nM Nfo for 2 h, lane 7, as 4 but 5 nM Nfo for 2 h; lane 8, as 4 but 1 nM Apn1 for 2 h; lane 9, as 4 but 5 nM Apn1 for 2 h, line 10, control, non-treated εA22•T; lane 11, 13 mer size marker; lane 12, as 10 but 1 nM Nfo for 2 h; lane 13, as 10 but 1 nM Apn1 for 2 h, lane 14, 14 mer size marker, line 15, control, non-treated εC22•T; line 16, as 15 but 1 nM Nfo for 2 h; lane 17, as 15 but 1 nM Apn1 for 2 h. For details see Materials and Methods. (TIF) [file pone.0051776.s002.tif]

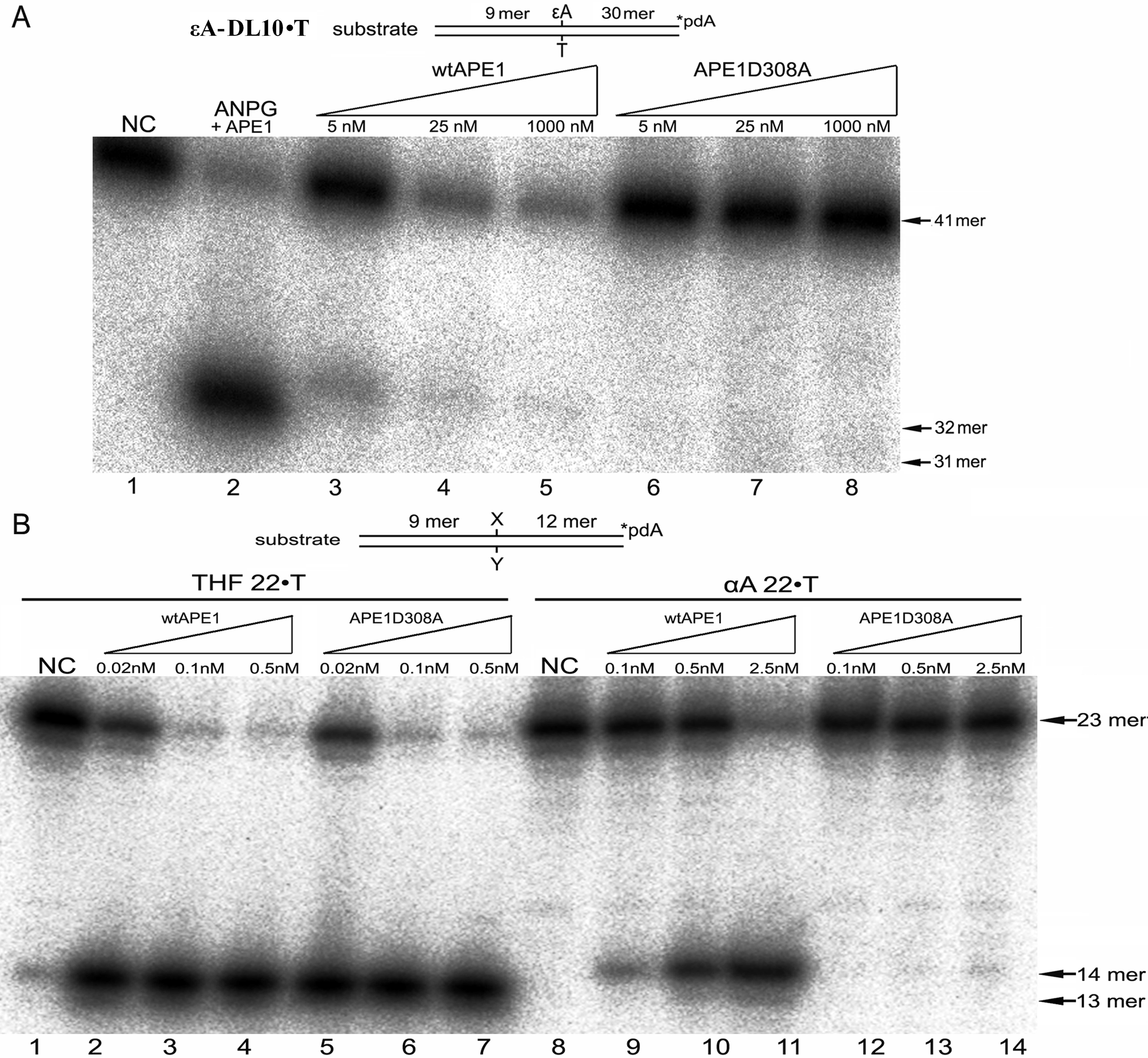

Supplement: Figure S3 — Comparison of NIR and AP endonuclease activities of APE1-WT and mutant APE1-D308A proteins. A solution of 10 nM of 22 mer 3′-[32P]-labelled εA22•T, THF•T and αdA•T oligonucleotide duplexes were incubated with varying amounts of the APE1 proteins under NIR conditions, and products of the reaction were analyzed using denaturing PAGE. (A) εA22•T duplex was incubated with varying amounts of the APE1 WT and APE1-D308A mutant proteins for 4 h at 37°C. Lane 1, control, non-treated εA22•T; lane 2, 20 nM ANPG and 10 nM APE1 under BER+Mg2+ conditions; lane 3, 5 nM of APE1-WT; lane 4, 25 nM of APE1-WT; lane 5, 100 nM of APE1-WT; lane 6, 5 nM of APE1-D308A; lane 7, 25 nM of APE1-D308A; lane 8, 100 nM of APE1-D308A. (B) THF•T and αdA•T duplexes were incubated with varying amounts of the APE1-WT and APE1-D308A mutant proteins for 5 min at 37°C. Lane 1, control, non-treated THF•T; lane 2, 0.02 nM of APE1-WT; lane 3, 0.1 nM of APE1-WT; lane 4, 0.5 nM of APE1-WT; lane 5, 0.02 nM of APE1-D308A; lane 6, 0.1 nM of APE1-D308A; lane 7, 0.5 nM of APE1-D308A; Lane 8, control, non-treated αdA•T; lane 9, 0.1 nM of APE1-WT; lane 10, 0.5 nM of APE1-WT; lane 11, 2.5 nM of APE1-WT; lane 12, 0.1 nM of APE1-D308A; lane 13, 0.5 nM of APE1-D308A; lane 14, 2.5 nM of APE1-D308A. For details see Materials and Methods. (TIF) [file pone.0051776.s003.tif]

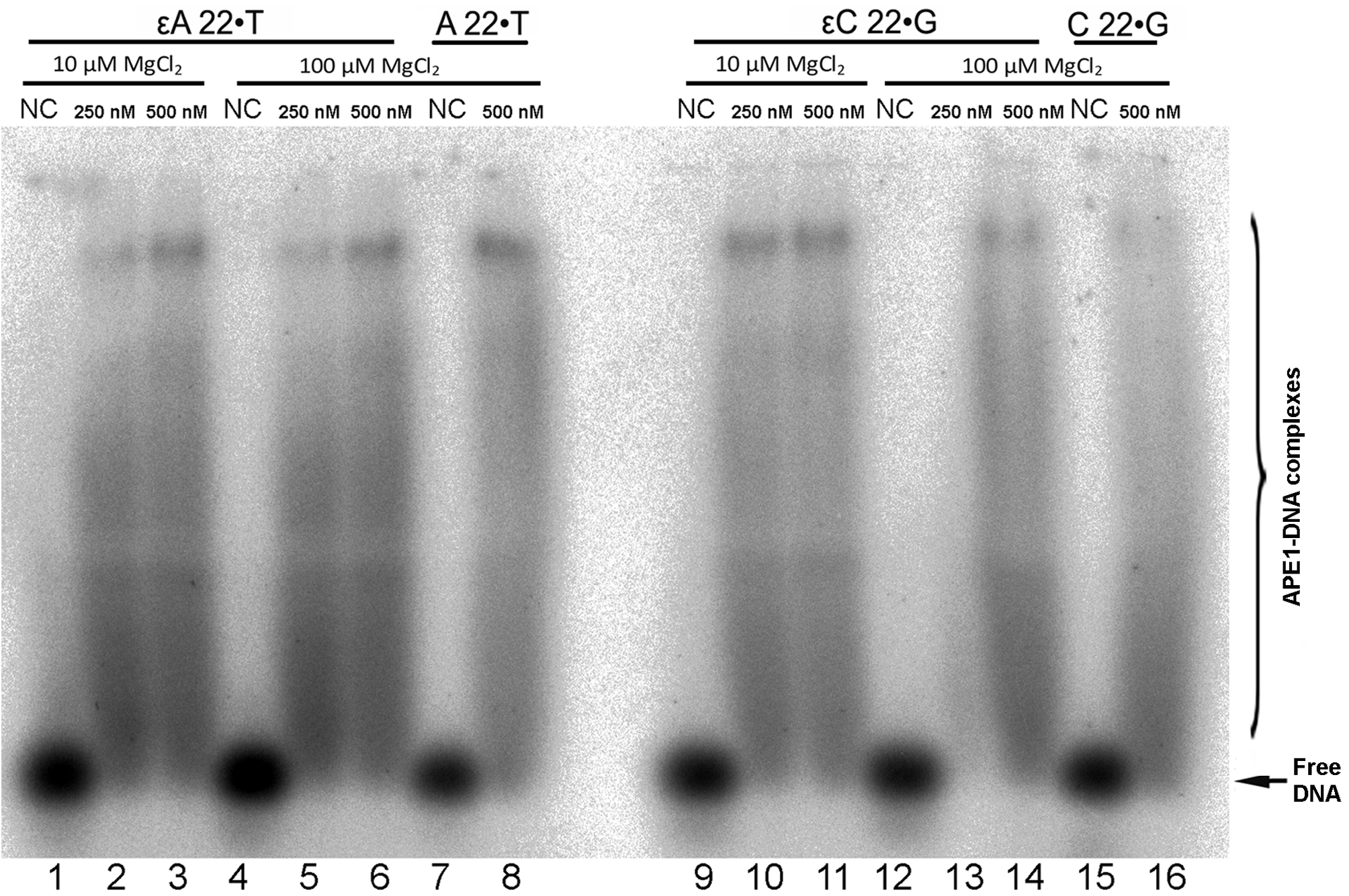

Supplement: Figure S4 — Electrophoretic Mobility Shift Assay (EMSA) for binding of APE1 to oligonucleotide duplexes containing a single ε-base. The standard binding reaction mixture (20 µl) contained 20 mM Hepes-KOH, pH 7.6, 50 mM KCl, 10 µM or 100 µM MgCl2, 10 nM of 22 mer 3′-[32P]-labelled εA22•T, A22•T, εC22•G or C22•G and 250 nM or 500 nM APE1. The mixture was incubated for 10 min on ice, after which an aliquot was analyzed by electrophoresis on a 8% non-denaturing polyacrylamide gel (19∶1 acrylamide/bisacrylamide) at 160V for 14 h at +4°C. Lane 1, control, εA22•T in 10 µM MgCl2; lane 2, as 1 but 250 nM APE1; lane 3, as 1 but 500 nM APE1; lane 4, control, εA22•T in 100 µM MgCl2; lane 5, as 4 but 250 nM APE1; lane 6, as 4 but 500 nM APE1; lane 7, control, A22•T in 100 µM MgCl2; lane 8, as 7 but 500 nM APE1; lane 9, control, εC22•T in 10 µM MgCl2; lane 10, as 9 but 250 nM APE1; lane 11, as 9 but 500 nM APE1; lane 12, control, εC22•T in 100 µM MgCl2; lane 13, as 12 but 250 nM APE1; lane 14. as 12 but 500 nM APE1; lane 15, control, C22•G in 100 µM MgCl2; lane 16, as 15 but 500 nM APE1. For details see Materials and Methods. (TIF) [file pone.0051776.s004.tif]

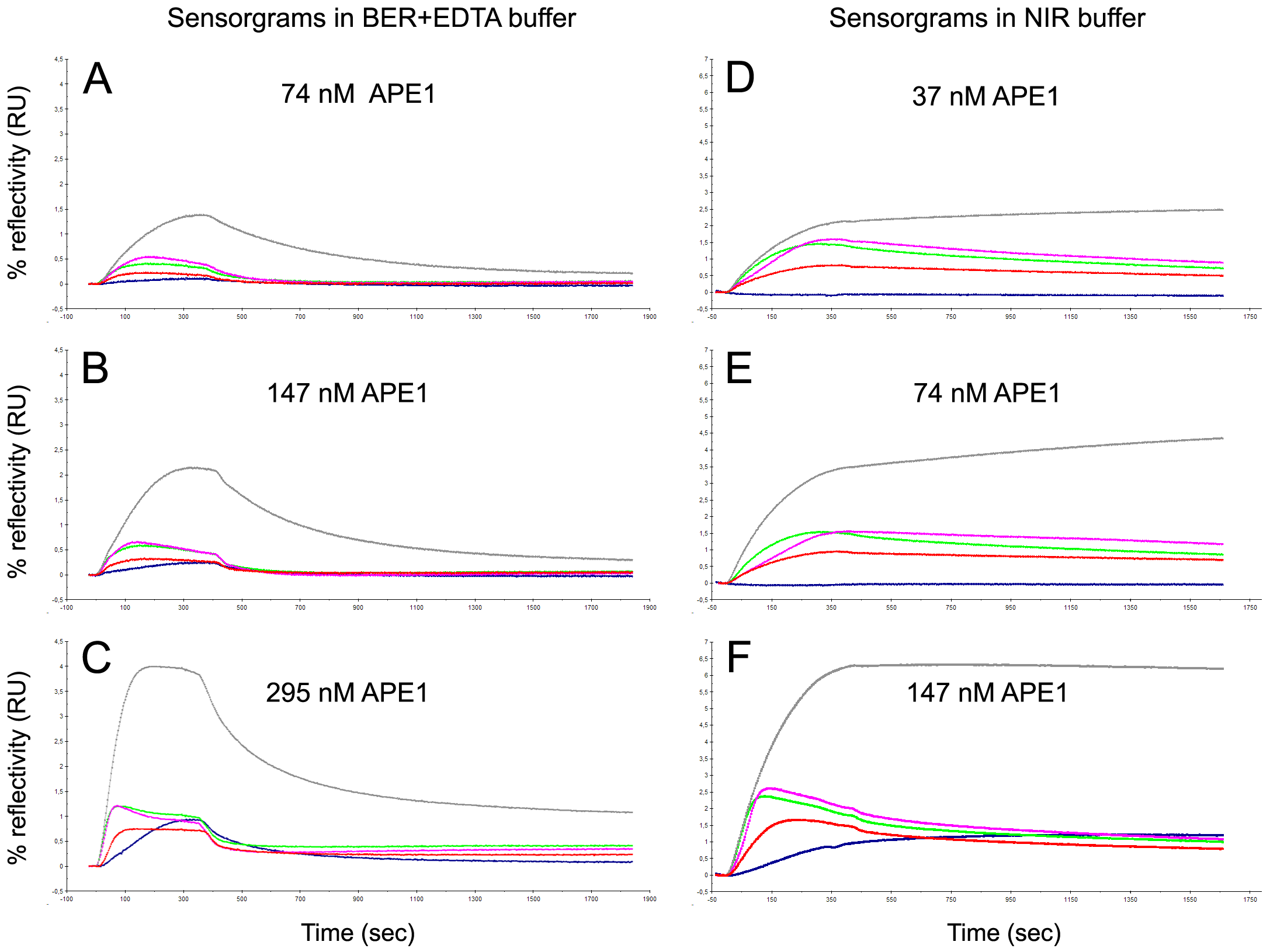

Supplement: Figure S5 — SPRi kinetic curves of APE1 (37–296 nM) interacting with immobilized DNA on the pre-treated surface. (A–C) Measurements were performed in “BER+EDTA” buffer. (D–F) Measurements were performed in NIR buffer. The curves representing the interactions of APE1 with Hairpin DNA (HP) are in blue, regular single-stranded T22 oligonucleotides are in grey, εA22•T duplexes are in green, THF-22•T duplexes in pink and A22•T duplexes in red. For details see Materials and Methods. (TIF) [file pone.0051776.s005.tif]

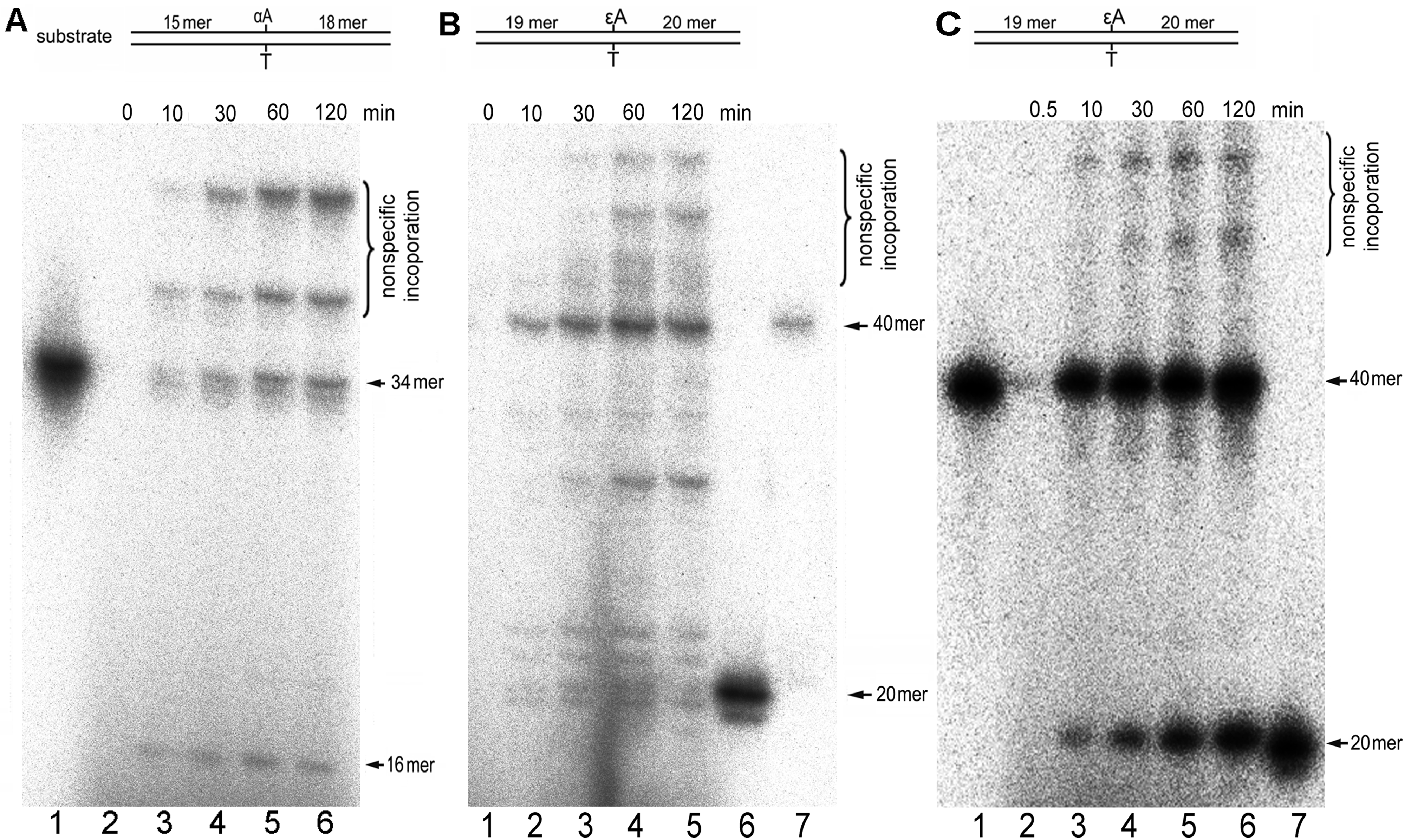

Supplement: Figure S6 — Time kinetics of in vitro reconstitution of the NIR and BER pathways using oligonucleotide duplex containing single αdA or εA residue. (A,B) A solution of 10 nM of non-labelled oligonucleotide duplexes was incubated at 37°C for various times up to 120 min in the presence of 10 nM APE1, 2 nM FEN1, 0.01 U POLβ and 4 U T4 DNA ligase in a reaction buffer containing 50 mM HEPES-KOH (pH 7.2), 30 mM NaCl, 3 mM MgCl2, 2 mM ATP, 0.1 mg/ml BSA, 2 mM DTT, 5 mCi of [α-32P]dATP and 50 µM each of dGTP, dCTP and TTP. (A) 34 mer αdA-PN•T duplex. Lane 1, 34 mer size marker; lane 2, 0 min of incubation; lane 3, 10 min; lane 4, 30 min; lane 5, 60 min, lane 6, 120 min; (B) 40 mer εA-PP•T duplex. Lane 1, 0 min of incubation; lane 2, 10 min; lane 3, 30 min; lane 4, 60 min; lane 5, 120 min; lane 6, 20 mer size marker; lane 7, 40 mer size marker. (C) 10 nM of non-labelled 40 mer εA-PP•T oligonucleotide duplex was incubated at 37°C for various times up to 120 min in the presence of 40 nM ANPG, 5 nM APE1, 2 nM FEN1, 0.01 U POLβ and 4 U T4 DNA ligase in the reaction buffer containing 20 mM HEPES-KOH (pH 7.6), 50 mM KCl, 5 mM MgCl2, 2 mM ATP, 0.1 mg/ml BSA, 1 mM DTT and 5 mCi of [α-32P]dATP. Lane 1, 40 mer size marker; lane 2, 0.5 min of incubation; lane 3, 10 min; lane 4, 30 min; lane 5, 60 min; lane 6, 120 min; lane 7, 20 mer size marker. For details see Materials and Methods. (TIF) [file pone.0051776.s006.tif]

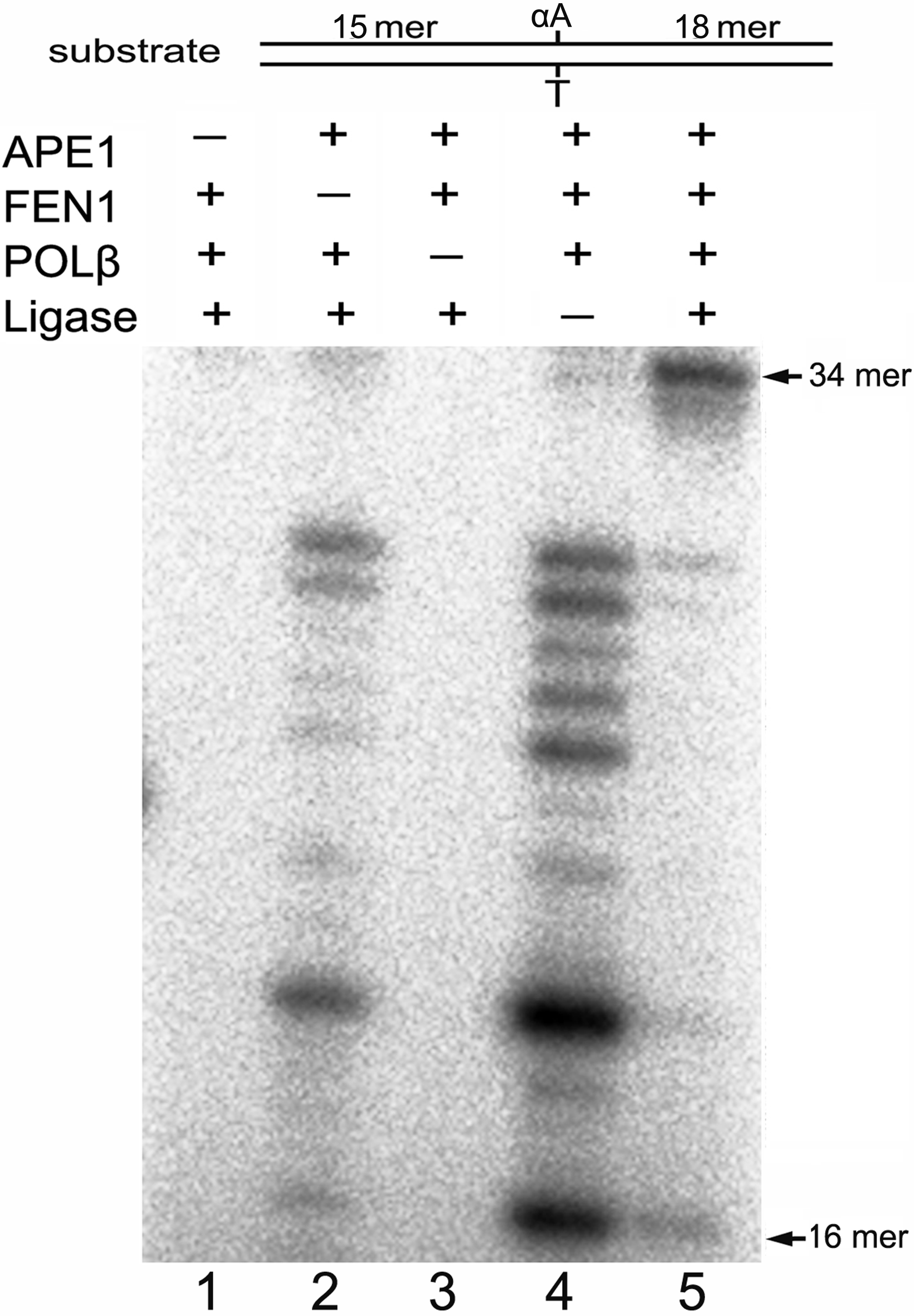

Supplement: Figure S7 — In vitro reconstitution of the long-patch NIR pathway using oligonucleotide duplex containing single αdA residue. 10 nM of non-labelled 34 mer αA-PN•T oligonucleotide duplex was incubated for 3 h at 37°C in the presence of 10 nM APE1, 2 nM FEN1, 0.02 U POLβ and 20 U T4 DNA ligase in the reaction buffer containing 50 mM HEPES-KOH (pH 7.2), 30 mM NaCl, 3 mM MgCl2, 2 mM ATP, 0.1 mg/ml BSA, 2 mM DTT, 5 mCi of [α-32P]dATP and 50 µM each of dGTP, dCTP and TTP. Lane 1, αA-PN•T incubated with all proteins except APE1; lane 2, except FEN1; lane 3, except POLβ; lane 4, except ligase; lane 5, in the presence of all proteins. The arrows denote the position of 16 mer cleavage product and 34 mer full-length product. For details see Materials and Methods. (TIF) [file pone.0051776.s007.tif]
