# Supplementary material for: Highly Mutagenic Exocyclic DNA Adducts Are Substrates for the Human Nucleotide Incision Repair Pathway
Source: PLoS One. 2012 Dec 14;7(12):e51776. doi: 10.1371/journal.pone.0051776 (PMC3522590; doi:10.1371/journal.pone.0051776)
Supplement: Table S1 — Kinetic constants for APE1-DNA substrate interactions deduced from sensorgrams using Bia evaluation software. (DOC) [file pone.0051776.s008.doc]

**Table S1**. Kinetic constants for APE1-DNA substrate interactions deduced from sensorgrams using Bia evaluation software.

| SPRi measurements performed in NIR buffer | | | | | |
| --- | --- | --- | --- | --- | --- |
| DNA substrate | APE1 (nM) | *k*_a_.(M^-1^s^-1^) | *k*_d_ (s^-1^) | *K*_d_ (M) | Mean *K*_d_ (M) |
| HP | 37 | ND | ND | ND | 16.4×10^-9^ |
|  | 74 | ND | ND | ND |  |
|  | 147 | 2.25×10^3^ | 3.68×10^-5^ | 1.64×10^-8^ |  |
| T22 (ssDNA) | 37 | ND | ND | ND | 6.72×10^-9^ |
|  | 74 | ND | ND | ND |  |
|  | 147 | 4.39×10^4^ | 2.95×10^-4^ | 6.72×10^-9^ |  |
| εA22•T | 37 | 2.44×10^5^ | 4.53×10^-4^ | 1.85×10^-9^ | 1.79±1.03×10^-9^ |
|  | 74 | 1.41×10^5^ | 3.94×10^-4^ | 2.79×10^-9^ |  |
|  | 147 | 3.18×10^5^ | 2.35×10^-4^ | 7.38×10^-10^ |  |
| A22•T | 37 | 1.52×10^5^ | 3.43×10^-4^ | 2.26×10^-9^ | 2.16±0.55×10^-9^ |
|  | 74 | 8.11×10^4^ | 2.15×10^-4^ | 2.65×10^-9^ |  |
|  | 147 | 1.65×10^5^ | 2.60×10^-4^ | 1.57×10^-9^ |  |
| THF-22•T | 37 | 1.79×10^5^ | 4.01×10^-4^ | 2.24×10^-9^ | 2.79±1.75×10^-9^ |
|  | 74 | 4.25×10^4^ | 2.02×10^-4^ | 4.75×10^-9^ |  |
|  | 147 | 2.06×10^5^ | 2.84×10^-4^ | 1.38×10^-9^ |  |
| SPRi measurements performed in BER+EDTA buffer | | | | | |
| HP | 74 | 6.37×10^3^ | 5.65×10^-3^ | 8.87×10^-7^ | 310±490×10^-9^ |
|  | 147 | 3.09×10^4^ | 1.26×10^-3^ | 4.08×10^-8^ |  |
|  | 295 | 1.1×10^4^ | 2.35×10^-4^ | 2.13×10^-8^ |  |
| T22 (ssDNA) | 74 | 2.3×10^5^ | 1.14×10^-3^ | 4.96×10^-9^ | 3.82±1.08×10^-9^ |
|  | 147 | 1.74×10^5^ | 6.42×10^-4^ | 3.69×10^-9^ |  |
|  | 295 | 3.35×10^4^ | 9.43×10^-5^ | 2.82×10^-9^ |  |
| εA22•T | 74 | 3.07×10^5^ | 9×10^-4^ | 2.93×10^-9^ | 1.89±1.48×10^-9^ |
|  | 147 | ND | ND | ND |  |
|  | 295 | 2.36×10^5^ | 1.98×10^-4^ | 8.41×10^-10^ |  |
| A22•T | 74 | 4.77×10^5^ | 1.13×10^-3^ | 2.37×10^-9^ | 1.76±0.86×10^-9^ |
|  | 147 | ND | ND | ND |  |
|  | 295 | 1.47×10^5^ | 1.69×10^-4^ | 1.15×10^-9^ |  |
| THF-22•T | 74 | 2.65×10^5^ | 1.01×10^-4^ | 3.81×10^-10^ | 0.94±0.79×10^-9^ |
|  | 147 | ND | ND | ND |  |
|  | 295 | 1.58×10^5^ | 2.37×10^-4^ | 1.5×10^-9^ |  |

ND, non-determined.
